# Exome Sequencing in an Admixed Isolated Population Indicates *NFXL1* Variants Confer a Risk for Specific Language Impairment

**DOI:** 10.1371/journal.pgen.1004925

**Published:** 2015-03-17

**Authors:** Pía Villanueva, Ron Nudel, Alexander Hoischen, María Angélica Fernández, Nuala H. Simpson, Christian Gilissen, Rose H. Reader, Lillian Jara, Maria Magdalena Echeverry, Clyde Francks, Gillian Baird, Gina Conti-Ramsden, Anne O’Hare, Patrick F. Bolton, Elizabeth R. Hennessy, the SLI Consortium, Hernán Palomino, Luis Carvajal-Carmona, Joris A. Veltman, Jean-Baptiste Cazier, Zulema De Barbieri, Simon E. Fisher, Dianne F. Newbury

**Affiliations:** 1 Human Genetics Program, Institute of Biomedical Sciences (ICBM), Faculty of Medicine, University of Chile, Santiago, Chile; 2 School of Speech and Hearing Therapy, Faculty of Medicine, University of Chile, Santiago, Chile; 3 Department of Child and Dental Maxillary Orthopedics, Faculty of Dentistry, University of Chile, Santiago, Chile; 4 Doctoral Program of Psychology, Graduate School, University of Granada, Granada, Spain; 5 Wellcome Trust Centre for Human Genetics, University of Oxford, Oxford, United Kingdom; 6 Department of Human Genetics, Radboud Institute for Molecular Life Sciences and Donders Centre for Neuroscience, Radboud University Medical Center, Nijmegen, the Netherlands; 7 Grupo de Citogenetica, Filogenia y Evolucion de las Poblaciones, Facultades de Ciencias y de Ciencias de la Salud, Universidad del Tolima, Ibague, Colombia; 8 Max Planck Institute for Psycholinguistics, Nijmegen, the Netherlands; 9 Donders Institute for Brain, Cognition and Behaviour, Radboud University, Nijmegen, the Netherlands; 10 Newcomen Centre, the Evelina Children’s Hospital, London, United Kingdom; 11 School of Psychological Sciences, University of Manchester, Manchester, United Kingdom; 12 Department of Reproductive and Developmental Sciences, University of Edinburgh, Edinburgh, United Kingdom; 13 Departments of Child & Adolescent Psychiatry & Social Genetic & Developmental Psychiatry Centre, Institute of Psychiatry, King’s College London, London, United Kingdom; 14 University Child Health and DMDE, University of Aberdeen, Aberdeen, United Kingdom; 15 UC Davis Genome Center, Department of Biochemistry and Molecular Medicine, School of Medicine, University of California Davis, Davis, California, United States of America; 16 Department of Oncology, University of Oxford, Oxford, United Kingdom; 17 Centre for Computational Biology, University of Birmingham, Edgbaston, United Kingdom; 18 St Johns College, University of Oxford, Oxford, United Kingdom; UCLA, United States of America

## Abstract

Children affected by Specific Language Impairment (SLI) fail to acquire age appropriate language skills despite adequate intelligence and opportunity. SLI is highly heritable, but the understanding of underlying genetic mechanisms has proved challenging. In this study, we use molecular genetic techniques to investigate an admixed isolated founder population from the Robinson Crusoe Island (Chile), who are affected by a high incidence of SLI, increasing the power to discover contributory genetic factors. We utilize exome sequencing in selected individuals from this population to identify eight coding variants that are of putative significance. We then apply association analyses across the wider population to highlight a single rare coding variant (rs144169475, Minor Allele Frequency of 4.1% in admixed South American populations) in the *NFXL1* gene that confers a nonsynonymous change (N150K) and is significantly associated with language impairment in the Robinson Crusoe population (p = 2.04 × 10–4, 8 variants tested). Subsequent sequencing of NFXL1 in 117 UK SLI cases identified four individuals with heterozygous variants predicted to be of functional consequence. We conclude that coding variants within NFXL1 confer an increased risk of SLI within a complex genetic model.

## Author Summary

Children affected by Specific Language Impairment (SLI) have unexpected problems learning to talk and understand language, despite developing normally in all other areas. This disorder runs in families but we do not understand how the genetic contributions work, or which genetic mechanisms might be important. In this paper, we study a Chilean population who are affected by a high incidence of SLI. Such populations may provide increased power to discover contributory genetic factors, under appropriate conditions. We identify a genetic change in the population that causes a change to a protein called NFXL1. This change is usually very rare but is found at a higher frequency than expected in our population, particularly in those people affected by SLI. We then looked at this gene in over 100 individuals from the UK affected by SLI and found four more changes that probably affect the protein. This is a higher number than we would expect by chance. We therefore propose that the *NFXL1* gene and the protein it encodes might be important in risk of SLI.

## Introduction

Language deficits form a central feature of many developmental disorders and account for a high number of pediatric referrals and statements of special educational need [1]. These language impairments often represent a secondary clinical feature of a more pertinent developmental disability such as Down syndrome, Autistic Spectrum Disorder or intellectual disability. However, in a proportion of cases, the primary clinical concern is the language difficulties, which occur in the absence of any other developmental deficit or neurological impairment and in the presence of normal non-verbal IQ. In such cases, the diagnosis is Specific Language Impairment (SLI) [2].

SLI affects between 5 and 7% of children in the UK [3] and significantly more boys than girls [4]. The disorder is highly heritable [5] but genetic contributions are expected to be complex in nature with significant heterogeneity between individuals [6]. Common risk variants within *ATP2C2* (OMIM#613082), *CMIP* (OMIM#610112) [7], *ABCC13* (OMIM#608835) [8], *FLNC* (OMIM#102565), *RBFOX2* (OMIM#612149) [9] and *ROBO2* (OMIM#602431) [10] have been associated with quantitative measures of language skills. Genome-wide association studies of language-impaired probands have also highlighted potential risk variants in *NDST4* (OMIM#615039), *ZNF385D, COL4A2* (OMIM#120090) *[11]* and *NOP9 [12]*. Other studies implicate rare genetic events which may have higher penetrance [13,14]. However, it is clear that the contributions of these various genetic effects are complex. Some may be specific to individuals with certain forms of language deficits, others may contribute across the range of ability [7,8,11,15,16]. The functional impact of these candidate genes has yet to be elucidated and further candidates need to be identified before we can properly understand the molecular pathways underlying SLI.

Clearer links have been made between the presence of language deficits and disruption of the *FOXP2* gene (OMIM#605317), a forkhead/winged-helix transcription factor [17,18]. Reduced functional dosage of *FOXP2*, caused by mutation or chromosomal rearrangements, leads to characteristic deficits in coordinating sequences of orofacial movements, impairing speech, producing a disorder known as developmental verbal dyspraxia (DVD) or childhood apraxia of speech (CAS) [18–22]. Typically the DVD/CAS features of *FOXP2* mutation cases are accompanied by wide-ranging problems with spoken and written language [23]. Whilst *FOXP2* disruptions are rare and account for only a small proportion of DVD/CAS cases, the investigation of this gene, its expression patterns and interactions, have led to the elucidation of genetic networks that are important to language development and contribute to more common forms of language impairment [23–25]. One of the transcriptional targets of FOXP2 is *CNTNAP2* (OMIM#604569), a member of the neurexin family which mediates interactions between neurons and glia during nervous system development [26]. Genetic variation across *CNTNAP2* has been associated both with language deficits [15,27–29] and language ability in the general population [30–32]. Variations in, and disruptions of, this gene have also been implicated across a range of neurodevelopmental disorders such as autism, epilepsy and schizophrenia [26], indicating that it is likely to be crucial for brain development. These investigations demonstrate how the identification of genetic mutations underlying a distinct severe form of disorder provide entry points into mechanisms that are relevant to the wider processes underlying the initial deficit.

In 2008, Villanueva et al described a population who are affected by an unusually high prevalence of language impairment [33]. This admixed population inhabits the Robinson Crusoe Island which forms part of the Juan Fernandez Archipelago in the South Pacific Ocean, approximately 400 miles off the coast of Chile. The Island was last colonized in 1876 by 64 individuals of European and South American descent. In the 2002 census, the Island population was 633, the majority of whom were descendants of the founder families. More than 70% of the current population has a surname from the colonizing families and 14% of marriages involve consanguineous unions [34]. In their 2008 study, Villanueva et al completed psychometric profiling of 66 island children aged between 3 and 9 years of age, of whom 40 were descendants of the founder party. They found that 35% of the founder-related children (14 of 40) were affected by specific language impairment. No evidence for a male bias was observed in this group. A further 27.5% of the founder-related child population (11 of 40) had language abilities below that expected for their age but presented with additional developmental concerns or low non-verbal IQ, precluding a diagnosis of SLI. The remaining 37.5% of founder-related children (15 of 40) had typical language development. In contrast, only one of 26 children whose parents are not related to the founder families (3.8%) had evidence of language impairment, a frequency of language impairment that coincided with that seen in mainland Chile (3%) [33]. Furthermore, when the genealogical records of the islanders were recompiled, 90% of the individuals affected by SLI were direct descendants of a single pair of founder brothers who formed part of the founder party [33,35]. Given the clear phenotypic differences between founder-related and non-founder-related children on the Island, we postulated that the founder brothers may have carried a rare causative genetic mutation or, alternatively, combinations of common genetic variations that together confer a high risk of language impairment. A previous genome-wide linkage study of 34 families from the Robinson Crusoe Island identified significant linkage to several chromosome regions, the most consistent of which included a large section (48Mb) of chromosome 7q (SLI4 – OMIM#612514) that included many genes which represent good candidates for language impairment, including *FOXP2* and *CNTNAP2* [35]. However, in depth genomic profiling has yet to be performed within this population.

In this study, we make use of this admixed isolated population and assess the possibility of a founder mutation, by completing exome sequencing of five individuals from the Robinson Crusoe population affected by SLI. We substantiate the findings of the exome screen by performing association analyses of selected putative functional variants in the wider Robinson Crusoe population. The contribution of identified risk variants is subsequently validated by performing targeted sequencing of candidate genes in a UK-based cohort of individuals affected by SLI.

## Results

**Fig 1:**
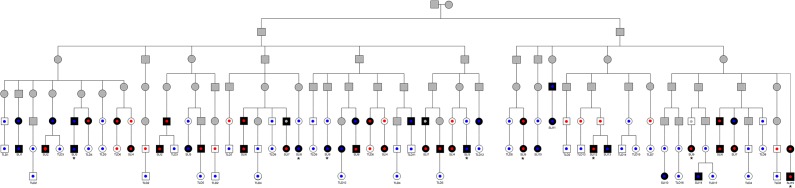
Pedigree showing direct lines of descent between founder brothers and children in Robinson Crusoe validation cohort. Founder brothers are individuals on the second line of the pedigree. Individuals with language impairment are colored in black. Individuals with typical language are denoted in white. Individuals with unknown phenotype are shaded grey. Genotypes at rs144169475 are represented by small circles; blue circles represent homozygote reference allele, red circles represent variant carriers, grey circles represent unknown genotype. Note that each individual may be represented through multiple lines of descent and so might appear more than once on this diagram. Children are labelled according to affection status—SLI1 to SLI15 and TLD1 to TLD17. Cases whose exomes were sequenced are indicated by asterisks. Three children (1 affected, 2 unaffected, none of whom carried the rs144169475 variant) are not represented on this figure since they were related to alternative founder families. SLI15 is known to be related to one of the founder brothers but the exact line of descent is unknown.

We selected five related individuals with SLI from the Robinson Crusoe cohort for exome sequencing (Fig. 1). From the exome sequence data, we selected all novel variants (i.e. not reported in publically available or in-house databases) that caused nonsynonymous changes or changes to canonical splice sites and were shared by at least three of the five individuals sequenced. A flow diagram of our methodology can be found in S1 Fig.. All such variants were subsequently genotyped in 111 founder-related cases and controls from the Robinson Crusoe Island (Robinson Crusoe validation cohort) and tested for association to language impairment using a method that takes into account familial relationships. To substantiate the findings of the exome screen and association analyses, we then went on to sequence the coding regions of candidate genes implicated from these investigations in an independent cohort of 117 British children affected by SLI (SLIC cohort).

### Exome sequencing

On average, 47,276 (median = 49,543, range = 43,075–50,112) genic variants were identified in each of the five exomes. This included an average of 17,405 (median = 17,326, range = 15,200–19,837) exonic variants, 8,379 (median = 8,089, range = 7,258–9,629) missense variants and 106 (median = 90, range = 72–157) nonsense (including indels) variants per individual. Across all five samples, 90.0% of targeted exome sequencing had coverage of at least 10-fold. The average coverage of targeted sequence was 56.5-fold and 21% of the reads reached this level. Sequencing metrics can be found in S1 Table. To test the hypothesis that the founder brothers carried a rare causative genetic mutation, we focused upon novel variants that caused nonsynonymous protein substitutions or altered canonical splice sites for our downstream analyses. Comparisons between individuals found that no such variants were shared by all five individuals. However, allowing for potential genetic heterogeneity between affected individuals, we identified nine novel nonsynonymous or splice-site variants that were shared by at least 3 of the 5 children sequenced (Table 1). Eight novel nonsynonymous or splice-site variants were validated in the five exome samples by Sanger sequencing. None of these variants overlapped with the regions of suggestive linkage (P<7.3×10^−4^, chromosomes 2, 6, 7, 8, 9, 12, 13 and 17, as listed in S2 Table) previously identified in this population [35]. S3 Table provides a full list of all shared, high-quality variants that fell within the previously identified regions of linkage. All of these had previously been reported in dbSNP (138) and many were non-genic, intronic or synonymous (see notes column in S3 Table).

**Table 1:** Association of novel nonsynonymous or canonical splice-site variants in 111 individuals from the Robinson Crusoe validation cohort.

Chr	Variant Position (hg19)	Ref/variant	Average read depth across variant	Gene	Transcript ID	Gene element affected by variant	Amino Acid change	SLI/TLD^1^	Variant Freq^2^	SLI variant freq^3^	TLD variant freq^4^	MQLS p
1	113,245,326	A/G	60	*RHOC*	NM_001042678	IVS3	SA site	49/62	0.059	0.071	0.048	0.625
1	248,308,783*	T/A	415	*OR2M5*	NM_001004690	Exon 1	C112S	49/62	0.000	0.000	0.000	-
4	47,907,320	A/T	57	*NFXL1*	NM_152995	Exon 4	N150K	49/62	0.113	0.194	0.048	0.0002
10	31,134,425	C/T	119	*ZNF438*	NM_001143766	Exon 8	R641H	49/62	0.158	0.173	0.145	0.466
11	33,054,503	T/G	36	*DEPDC7*	NM_139160	Exon 8	N444K	40/60	0.131	0.149	0.117	0.399
16	27,363,901	G/A	30	*IL4R*	NM_000418	Exon 7	R185H	49/61	0.095	0.143	0.057	0.053
21	47,359,924	C/T	52	*PCBP3*	NM_001130141	IVS-12	SA site	48/59	0.266	0.292	0.246	0.228
22	41,257,834	T/TA	37	*DNAJB7*	NM_145174	Exon 1	V55VX	49/62	0.261	0.245	0.274	0.554
X	48,682,972	A/G	30	*HDAC6*	NM_006044	Exon 29	N1200D	49/62	0.419	0.378	0.452	0.456

1 – The number of individuals with SLI genotyped / the number of individuals with typical language ability genotyped.

2 – Frequency of discovered variant in all genotyped Islanders

3 – Frequency of discovered variant in genotyped Islanders with SLI

4 – Frequency of discovered variant in genotyped Islanders with typical language ability

Note that all Islanders (both cases and controls) were related

*- this variant was not validated with Sanger sequencing and represents a false positive finding from the exome sequencing

### Association analyses of key variants in Robinson Crusoe validation cohort

All shared novel nonsynonymous or splice-site variants identified in the exome screen were subsequently genotyped in 111 members of the Robinson Crusoe population (49 individuals with language-impairment and 62 individuals with typical language ability). This validation cohort was ascertained via 35 children living on the Robinson Crusoe Island who had been diagnosed with SLI or who showed typical language development (as described in methods) and included the five children used in the exome sequencing. All children were descendants of the founder families of the Robinson Crusoe Island and, as such, the cases and controls used in these association analyses were inter-related (Fig. 1). We therefore employed an association algorithm that allowed for relatedness between cases (MQLS, [36]), and that took into account the shared ancestry of the Robinson Crusoe validation cohort (288 individuals over 5 generations). These analyses highlighted one particular coding variant (chr4:g.47,907,320A>T, hg19) that was present at a significantly higher frequency in Islanders with language impairment than in Islanders with typical language ability (Table 1). Thirty nine percent of Islanders with language impairment were found to carry this variant compared to ten percent of Islanders with typical language skills (p = 2.04 × 10^−4^) (Table 1). Across the Robinson Crusoe validation cohort, the minor allele frequency was 11.3% (25 of 222 chromosomes sampled) (Table 1).

### Predicted functional effects of chr4:g.47,907,320A>T

**Fig 2:**
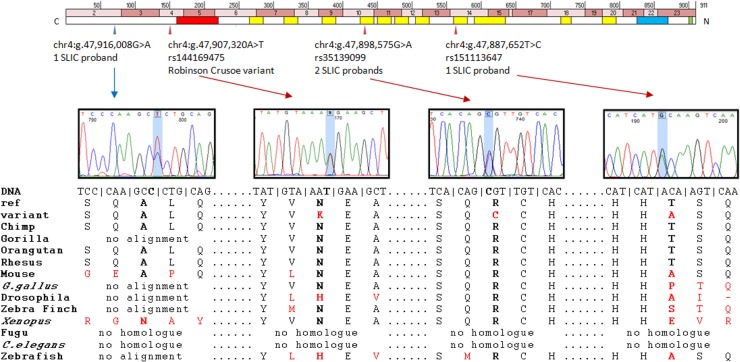
Putative contributory coding variants identified in *NFXL1* by this study. Position of putative *NFXL1* coding variants with respect to exons and protein coding sequence. Genomic coding exons (exons 2–23) are shown by pink bands at the top. Protein motifs are represented by colored bands in the lower boxes. The red box represents a Znf RING motif, the yellow boxes represent Znf NFX1 motifs, the blue box represents a coiled-coil domain and the green box a transmembrane domain. Putative contributory coding variants are shown by arrows. Blue arrows denote synonymous changes, red arrows nonsynonymous changes. Sanger sequencing plots are given for all variants identified. Conservation of amino acid sequences across 11 species shown for all variants identified. The ref row shows the human reference allele and the variant row shows the observed variant in our samples. All sequences that differ from the reference sequence are shown in red.

Chr4:g.47,907,320A>T (hg19) falls in exon 4 of the *Homo sapiens* nuclear transcription factor, X-box binding-like 1 (*NFXL1*) gene (Fig. 2). The variant causes a nonsynonymous change yielding an asparagine to lysine substitution in the encoded protein (p.N150K, uncharged amino acid to positively charged amino acid). This change is predicted to be “disease-causing” by MutationTaster with a confidence probability of 0.98 (SIFT = 0.67, PolyPhen-2 = 0.178). The position is conserved at both the amino acid and nucleotide level (PhyloP = 0.66, PhastCons = 1); the amino acid N150 is invariant across 36 of the 38 vertebrate species in which an alignment could be made and the thymine nucleotide at this position is conserved across all six ENSEMBL primate species investigated (Human, chimp, gorilla, orangutan, macque and marmoset) (Fig. 2).

### Chr4:g.47,907,320A>T, hg19 in control populations

The variant at chr4:47,907,320 was not observed in 127 independent European population controls that were genotyped (Table 2). We therefore went on to genotype an additional 320 independent individuals from a Colombian population cohort and 121 independent individuals from a Chilean control population cohort. In these cohorts, the variant was present with a minor allele frequency of 4.2% (27 of 640 chromosome sampled) and 7.4% (18 of 242 chromosome sampled) respectively (Table 2). Subsequent data released by the 1000 genomes project confirmed that this variant is specific to admixed American populations (AMR) with an average minor allele frequency of 4.1%. In the sub-populations of the AMR grouping, the minor allele frequency is reported as 0.9% in Puerto Ricans (PUR – 1 in 110 chromosomes sampled), 3.3% in Colombians in Medellin (CLM – 4 in 120 chromosomes sampled) and 7.6% in individuals of Mexican ancestry in Los Angeles (MXL – 10 of 132 chromosomes sampled) (Table 2). The variant has recently been designated as rs144169475 accordingly.

**Table 2:** Allele and genotype frequencies of rs144169475 in the Robinson Crusoe validation cohort.

	Robinson Crusoe population	Founder-related Islanders^1^	Non-founder-related Islanders^2^	SLI^3^	TLD^4^	Male Islanders^5^	Female Islanders^6^	European controls^7^	Colombian Controls^8^	Chilean Controls^9^	PUR^10^	CLM^11^	MXL^12^
freq allele T (variant)	0.113	0.125	0.000	0.194	0.048	0.116	0.132	0.000	0.042	0.074	0.009	0.033	0.076
freq genotype TT	0.000	0.000	0.000	0.000	0.000	0.000	0.000	0.000	0.000	0.000	0.000	0.000	0.000
freq genotype AT	0.225	0.250	0.000	0.388	0.097	0.233	0.263	0.000	0.084	0.149	0.018	0.067	0.152
freq genotype AA	0.775	0.750	1.000	0.612	0.903	0.767	0.737	1.000	0.916	0.851	0.982	0.933	0.848
No. individuals	111	100	11	49	62	43	57	127	320	121	55	60	66

1 – Islanders who are directly related to one of the eight founder families (NB this sample includes affected andunaffected individuals)

2 – Individuals who live on the Island but have no known genetic connection to the eight founder families (NB this sample includes 4 affected and 7 unaffected individuals)

3 – Islanders who have been diagnosed with SLI as described in methods (NB, this sample included 45 related, founder-related individuals and 4 non-founder-related parents).

4 – Islanders who have been classified as having typical language ability as described in methods (NB, this sample included 55 founder-related Islanders and 7 non-founder-related parents).

5 – Male individuals who are directly related to one of the eight founder families (NB this sample includes affected and unaffected individuals)

6 – Female individuals who are directly related to one of the eight founder families (NB this sample includes affected and unaffected individuals)

7 - 127 in-house European controls (ECACC, HRC-1 DNA Panel)

8 - 320 South American (Colombian) controls (provided by Luis Carvajal-Carmona and Maria Magdalena Echeverry)

9 – 121 Chilean controls (provided by Lillian Jara and Pia Villanueva)

10 – 1000 genomes Puerto Ricans from Puerto Rico (Integrated phase I, accessed March 2014)

11 – 1000 genomes Colombians from Medellin, Colombia (Integrated phase I, accessed March 2014)

12 – 1000 genomes Mexican Ancestry from Los Angeles USA (Integrated phase I, accessed March 2014)

### Linkage analyses of chromosome 4 (46–49Mb)

Parametric and nonparametric linkage analyses were performed for 55 SNPs across the *NFXL1* region of chromosome 4 (46–49Mb, hg19) within seven extended pedigrees from the Robinson Crusoe validation cohort (S2 Fig.). In these analyses, we did not observe evidence of linkage (maximum LOD score = 0.62, S3 Fig.).

### Sequencing of *NFXL1* in a language-impaired cohort (SLIC)

We sequenced the entire coding region of the *NFXL1* gene in 117 unrelated probands affected by SLI (from the UK SLI Consortium (SLIC) cohort [7,37–39]), to assess whether we could replicate a role for *NFXL1* in SLI etiology. In total, we identified 166 high-quality sequence variants across the *NFXL1* gene. 155 of the variants detected were intronic, 4 were in the 3’UTR and 7 affected the coding region. Of the coding variants, three were nonsynonymous and four were synonymous substitutions (Table 3).

**Table 3:** *NFXL1* coding variants observed in 117 UK (SLIC) probands affected by SLI.

Position (hg19)	Ref	Var	Estimated VAF in SLI probands^1^	Median read depth^2^	dbSNP ID	1000G population VAF (ALL:AFR:AMR:ASN:EUR)^3^	EVS VAF (EA:AA)^4^	European VAF^5^	Confirmed VAF in SLI probands^6^	*NFXL1* Exon	Amino Acid change^7^	Fishers exact between European controls & SLIC^8^
Chr4:g.47887536	T	C	0.7763	4531	rs2053404	0.73:0.65:0.75:0.72:0.77	0.75:0.68	0.75	NT	14	A601A	NT
Chr4:g.47887652	T	C	0.0035	5123	rs151113647	0.00:0.00:0.00:0.00:0.00	0.00:0.0002	0.00	0.0043	14	T563A	0.0244
Chr4:g.47887991	G	A	0.7835	6433.5	rs6818556	0.73:0.65:0.75:0.72:0.77	0.75:0.68	0.75	NT	13	T523T	NT
Chr4:g.47898575	G	A	0.0071	4817.5	rs35139099	0.0005:0.00:0.00:0.00:0.0013	0.005:0.0005	0.004	0.0085	10	R432C	ns
Chr4:g.47901088	C	T	0.0642	2986	rs34323060	0.02:0.002:0.03:0.00:0.04	0.047:0.0098	0.05	NT	7	K292K	NT
Chr4:g.47901476	G	A	0.3195	1212.5	rs12651301	0.42:0.33:0.35:0.63:0.35	0.31:0.33	0.31	NT	6	P246L	NT
Chr4:g.47916008	G	A	0.0071	2576.5	NA	0.00:0.00:0.00:0.00:0.00	0.00:0.00	0.00	0.0043	2	A71A	0.0244

1 – Variant allele freq (VAF) in 117 UK SLIC probands is estimated by Syzygy using the proportion of reads that have the variant

2 – Median read depth for given base across all pools

3 - Variant allele frequency (VAF) in 1000 genomes super-populations (Integrated phase I data, accessed March 2014). ALL – all 1000 genomes populations combined (No. alleles ∼ 2184), AFR – African populations (YRI, LWK, GWD, MSL, ESN, ASW & ACB, No. chromosomes = 492), AMR – Ad mixed Americans (MXL, PUR, CLM, PEL, No. chromosomes = 362),ASN – East Asian (CHB, JPT, CHS, CDX & KHV, No. chromosomes = 572), EUR-European (TSI, FIN, GBR, IBS, no. chromosomes = 758).

4 – Exome Sequencing Project (ESP) variant allele frequency (VAF). EA – European Americans (no. chromosomes = 8600), AA – African Americans (no. chromosomes = 4268).

5 – Combined variant allele frequency across European controls from 1000 genomes and EVS (no. chromosomes = 9358)

6 – Allele frequency in SLI probands after confirmatory Sanger sequencing (no. chromosomes = 234)

7 – Amino acid change conferred by given sequence variant in protein NP_694540.3. If the change occurs within a conserved motif, this is noted.

8 – Fisher’s exact test for differences in allele frequencies between EVS European Americans and SLIC probands. ns = non-significant P<0.05

NT = not tested

Ns = not significant

Nonsynonymous variants and those with estimated allele frequencies of <5% were verified across all the pools of DNA in which they were observed using Sanger sequencing. This allowed the derivation of accurate allele frequencies within the SLIC cohort.

One of the synonymous variants (chr4:g.47,916,008G>A, hg19) was found in a heterozygous state in one SLIC proband (allele frequency of 0.43%) but had not been documented in any European individuals in the 1000 genomes project [40] or the NHLBI GO ESP Exome Variant Server (EVS), which together consist of data from 4679 control individuals and therefore have the ability to detect rare variants with a population frequency of 0.0001. A comparison of allele frequencies between SLIC probands (1 of 234 chromosomes tested) and controls (0 of 9358 chromosomes tested) yielded a significant *P*-value of 0.0244. Intriguingly, although it is synonymous, this variant was predicted to be “disease-causing” by MutationTaster with a confidence probability of 0.98 (SIFT = 1.0). This variant falls in the most 5’ coding exon of *NFXL1* and is part of a CpG island, indicating that it may be important for the regulation of gene expression. Furthermore, ENCODE data shows that it is part of a H3K4Me3 mark (which is often associated with promoters) and binds multiple transcription factors, particularly POLR2A c-MYC and PHF8 (www.genome.ucsc.edu, accessed April 2014).

The remaining three synonymous variants (rs2053404, rs6818556 and rs35139099) found in SLIC probands were also found at similar frequencies in control databases. All had allele frequencies of >5% and are therefore thought to represent common polymorphisms (Table 3).

One nonsynonymous substitution (chr4:g.47,887,652T>C, hg19 – rs151113647) was found in a heterozygous state in a single SLIC proband (allele frequency of 0.43%) and again, was not observed in 4679 independent European individuals in the control public databases (Table 3), yielding a significant *P*-value of 0.024 (1 of 234 SLIC chromosomes tested vs 0 of 9358 control chromosomes tested). Further investigations found that this variant had been observed in a heterozygous state in a single African American individual from the EVS. Principal components analysis of genome-wide SNP data in the SLIC proband against the hapmap-3 populations did not detect any African ancestry. The rarity of the rs151113647 variant and its position within a zinc-finger motif (Fig. 2) indicates that it may confer negative effects upon protein function. Nonetheless, because the nucleotide is not highly conserved across species (phyloP = −0.418, phastCons = 0.925), the change was predicted to be a polymorphism by MutationTaster with a confidence probability of 0.99 (SIFT = 0.68, polyphen-2 = 0.00) (Fig. 2).

A second nonsynonymous substitution (chr4:47,898,575G>A, hg19 - rs35139099) was observed in a heterozygous state in two independent SLIC probands (allele frequency of 0.85%). This variant was also found in 44 of 4679 independent European control individuals from public databases (allele frequency of 0.47%, Table 3) yielding a P value of 0.3097. Although, it was not observed to occur at a significantly increased frequency in SLIC probands, the rs35139099 variant occurs at a conserved residue (phyloP = 1.466, phastCons = 1) within a zinc-finger motif (Fig. 2) and is therefore predicted to be damaging by MutationTaster with a confidence probability of 0.99 (SIFT = 0.00, Polyphen-2 = 1.00) (Fig. 2).

The remaining nonsynonymous variant (chr4:g.47,901,476G>A, hg19 - rs12651301) was observed to occur across all the sequence pools with an estimated allele frequency of 32% (Table 3). This common variant was also observed in independent European controls from public databases with a frequency of 31% (Table 3) and falls outside of any protein motifs and is thus likely to represent a polymorphism.

**Fig 3:**
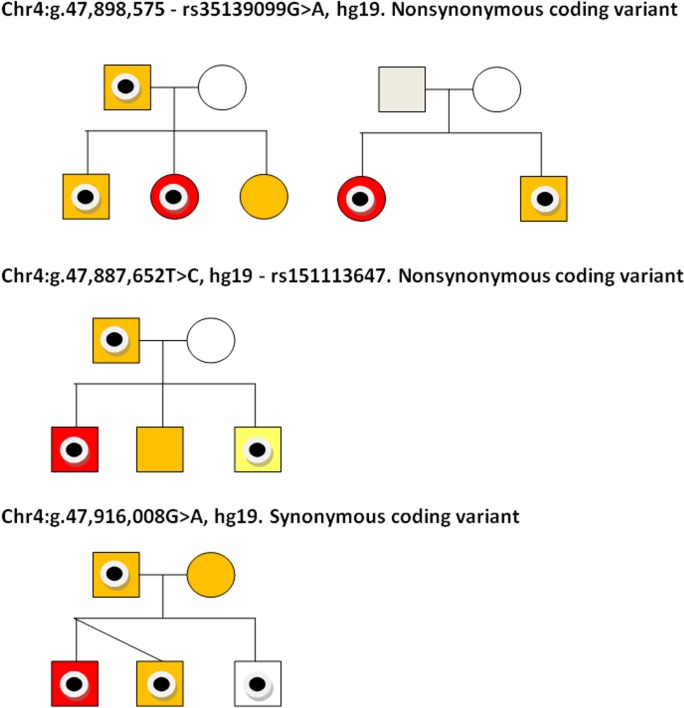
Coding variants observed in SLIC probands and their families. Pedigrees are shown for nuclear families of SLIC individuals carrying three coding variations in *NFXL1*. Individuals carrying the variants are identified with a black circle. Sequencing traces of each variant is shown. SLIC probands are colored in red and other family members with SLI (defined as expressive and/or receptive language skills >1.5SD below that expected for their age) are colored in orange. In pedigree 3 (rs151113647), the youngest sibling (colored in yellow) did not meet the criteria for SLI but had expressive and receptive language scores ∼1SD below that expected for his age. Individuals with no shading have typical language ability. DNA was not available for individuals colored in grey.

The three rare variants identified (rs151113647, rs35139099 and chr4:g.47,916,008G>A, hg19) were sequenced in all available family members of the SLIC proband in whom they were observed (Fig. 3). The chr4:g.47,916,008 variant was inherited from an affected father by two affected children and one child with typical language development (Fig. 3). The rs151113647 variant was inherited from a father, who reports a history of language and literacy problems, by the proband, who attends a special language unit, and his sibling, who also has SLI. The middle child in this family, who also showed evidence of expressive and receptive language deficits, did not inherit the variant (Fig. 3). Two SLIC families carried the rs35139099 variant; in the first, the variant is present in the father, who self-reports a history of dyspraxia, and passed onto both the proband and her elder sib, each of whom has expressive and receptive language problems. The youngest daughter in this family, who was observed to have a similar pattern of language deficits, did not inherit the variant (Fig. 3). In the second family carrying the rs35139099 variant, the change was present in both the proband and his younger sib, who had expressive and receptive language scores ∼1SD below that expected for his age, indicating that it is inherited (Fig. 3). The variant was not present in the mother and we did not have a DNA sample, or phenotypic data, from the father. Nonetheless, haplotype analyses of genome-wide SNP data indicated that the two children shared the same paternal chromosome in this region indicating that the rs35139099 variant was likely inherited from the father.

## Discussion

In this paper, we report results from the whole exome sequencing of five individuals from an isolated Chilean island population affected by a high incidence of SLI. We identify a heterozygous nonsynonymous coding variant in the *NFXL1* gene that is shared by three of the five individuals sequenced. Association analyses within a larger Robinson Crusoe validation cohort, demonstrated that this variant occurred at a significantly increased frequency in Islanders with language impairment than those with typical language development (P = 0.0002) and is predicted to be “disease-causing”. Subsequent sequencing of *NFXL1* in a cohort consisting of 117 independent UK probands (SLIC) with SLI identified four individuals with putative high-risk variants in the heterozygous state; three SLIC individuals carried rare nonsynonymous changes and one SLIC individual carried a novel variant that falls within a regulatory motif. Given the above evidence, we postulate that variants within *NFXL1* may contribute to genetic risk of language impairment. We propose that such changes are likely to function as—risk variants with a complex model of inheritance.

We used the Robinson Crusoe ancestry to trace back the relationships between individuals carrying the associated rs144169475 variant. The only common ancestors to the carriers were two founder brothers who had previously been reported to head the SLI lineage on the Island (Fig. 1). These brothers were related to all carriers of the rs144169475 variant (Fig. 1). However no single brother was related to all Islanders carrying the variant allele (Fig. 1). We therefore concluded that both founder brothers are likely to have carried the variant. These data therefore support the founder model of language impairment proposed at the outset of this study. We performed allele dropping simulations within the descendants of these founder brothers and found that a variant with an allele frequency of 3–9% in the founder population would be expected to have a frequency of 8–14% in the current population (S1 Text, S4 Table). This prediction fits well with the observed frequency of 12.5% in the founder-related Islanders and is elevated above that observed in Chilean population controls (7.4%), indicating the presence of a founder effect at this locus. Moreover, we found that the increased frequency of the rs144169475 variant is driven by Islanders with SLI (19.4% in 49 individuals with SLI vs 4.8% in 62 individuals with typical language ability) (Table 2).

Our data further suggest that the effects of rare mutations in *NFXL1* may extend to the etiology of SLI in other populations. In a screen of the *NFXL1* coding regions in 117 independent UK probands affected by SLI, we observed four individuals who carried rare coding changes generating a combined high risk allele frequency of 1.71%. By contrast, the combined allele frequency of these three variants in 4679 independent European controls (from the 1000 genomes and EVS public databases) is 0.47%, a difference that yields a marginally significant *P*-value of 0.029 (4 of 234 SLIC chromosomes vs 44 of 9358 control chromosomes). Extending our investigations to include all private coding mutations (i.e. only found in one individual) across the entire *NFXL1* transcript, as opposed to the consideration of the three specific mutations considered above, we again observed a marginally increased frequency in the SLIC cohort (2 of 234 chromosomes tested, 0.85%) above that expected given the data reported in public European databases (EVS European American and 1000 Genomes EUR super-population – 28 of 9358 chromosome sequenced, 0.3%, P = 0.0359). Broadening our investigation to include all rare coding changes (<1%) across the entire *NFXL1* transcript, revealed a similar trend (1.71% (4 of 234 chromosomes sequenced) in the SLIC cohort, compared to 1.36% (127 of 9358 chromosome sequenced) in public European databases) but this did not reach significance (P = 0.3821).

Given our consistent findings across cohorts, and in line with the data arising from other neurodevelopmental disorders, we suggest that rare variants in *NFXL1* may represent genetic risk factors with incomplete penetrance. Given our data, it is likely that these putative risk factors are modulated by other genetic variations and/or environmental factors [41–43]. We could not identify a distinct or specific phenotypic feature that distinguished rs144169475 language-impaired carriers from language-impaired non-carriers. Nor did we observe complete co-segregation between *NFXL1* variants and the presence of SLI in either the Robinson Crusoe validation population or the UK SLIC cohort. Thirty nine percent of the Robinson Crusoe validation cohort affected by language impairment carried the rs144169475 variant, as did ten percent of the Robinson Crusoe validation cohort with typical language ability. Similarly, one of the variants observed in the SLIC probands was inherited by a child with typical language development and two children affected by language impairment did not inherit the observed variant. In addition, we observed a high phenocopy rate in the Robinson Crusoe cohort; only 39% of individuals affected by language impairment carried the rs144169475 variant. Incomplete segregation is commonly described in neurodevelopmental disorders such as autism [42,43] and intellectual disability [44,45] and represents a major challenge in the interpretation of high-throughput sequencing data [46].

The *NFXL1* gene encodes a NFX-1-type nuclear zinc-finger transcriptional repressor that is expressed at the cytoplasm [47]. Little is known regarding the function of the NFXL1 protein; no disorders have been identified that arise from the mutation of this gene and no animal knock-outs have been described. The protein has zinc-finger domains which mediate DNA binding and carries a RING domain that has E3 ubiquitin ligase function (Fig. 2) [48]. This transcription factor has been shown to be highly expressed in embryonic stem cells prior to differentiation into myelinated oligodendrocytes [49] and shows a high level of expression in the early mouse embryonic development (E11.5) and in human cerebellar structures (www.brainmap.org). *NFXL1* is so-called because it is a paralogue of the NF-X1 transcription factor which binds the X-box sequence of class II *MHC* genes [50]. This feature may be relevant in light of a recent study that found association between HLA loci and SLI [51]. Similarly, an *NF-X1* isoform functions in the regulation of the NFĸB pathway [52], as does *CMIP*, a gene implicated in the etiology of SLI in UK populations [7,53].

### Limitations of our study

A natural limitation of all studies of founder or isolated populations is the restricted size of the cohort. Although our study represents a comprehensive profiling of the Robinson Crusoe child population, the total sample consisted of only 111 individuals, 100 of whom were founder-related and 49 of whom had language impairment. Although it should be noted that the power of this particular sample lies in the close relationships between individuals rather than the absolute number of samples, the issue of sample sizes is especially pertinent when one is considering rare variations. Thus it is of particular importance that we observed independent evidence implicating NFXL1 rare variants in another cohort. However, in the absence of a large South American cohort of language-impaired individuals, we were unable to include the rs144169475 variant in our replication investigations (since this SNP is particular to South American populations). Thus, further studies of larger sample sizes that include language-selected controls and South American individuals will be required to fully evaluate the role of rs144169475 and rare *NFXL1* coding variants in SLI susceptibility.

Of note, none of the shared variants identified through exome sequencing co-incided with regions of suggestive linkage reported in a previous genomewide linkage study of the Robinson Crusoe population (S2 and S3 Tables) [54]. Nor did we find evidence for linkage to the *NFXL1* region of chromosome 4 (S4 Fig.). We must therefore acknowledge that the increased frequency of rs144169475 in language-impaired individuals of the Robinson Crusoe validation cohort does not directly indicate pathogenicity. The result may represent a chance finding or, alternatively, rs144169475 may be a proxy for the causal variant. Since the exome sequencing performed did not capture 100% of the exome, it is possible that the causal variant was not detected here. Full genome sequencing would be required to fully investigate this possibility. However, it is also important to note that a lack of linkage does not preclude the presence of a causal variant and may instead reflect the complexities of analyzing a pedigree of this size and complexity [55]. The pedigree, which explained the known relationships between the founder brothers and the Robinson Crusoe validation cohort, included 288 individuals (321 bits, where a bit is defined as twice the number of non-founders—the number of founders) and so had to be broken into smaller sets for linkage analyses. This segmentation process discards information and can reduce the power to detect linkage if individuals sharing the linked chromosome segment are split between sub-pedigrees [56]. Lastly, since we hypothesize that SLI in this population has a complex genetic basis and involves incomplete and a high phenocopy frequency, it is possible that the power to detect linkage is insufficient. We observed reduced penetrance at the *NFXL1* locus (of 25 variant carriers, 19 were diagnosed with SLI, penetrance of 76%) in combination with evidence of a high phenocopy rate in our cohort (of 49 individuals with language impairment, 19 carried the variant, phenocopy rate of 61%). In combination, these factors break down the correspondence between genotype and phenotype, compromising the ability to detect linkage [57].

In summary, the Robinson Crusoe admixed founder population represents a rare resource which may assist in the identification of genetic variants that contribute to SLI susceptibility. Exome sequencing of five individuals from this population identified eight shared coding variants. One of these variants (rs144169475) was found to be significantly associated (P = 0.0002) with language impairment in the wider Robinson Crusoe population. rs144169475 confers a nonsynonymous change (N150K) in the *NFXL1* gene at a highly conserved residue. Subsequent sequencing of the *NFXL1* coding regions in 117 independent UK SLI cases identified four individuals with rare heterozygous variants predicted to be of functional consequence. We conclude that coding variants within *NFXL1* confer an increased risk of SLI within a complex genetic model.

## Materials and Methods

### Ethics

The work on the Robinson Crusoe Island was approved by the ethics department of the University of Chile. Ethical permission for each SLIC collection was granted by local ethics committees. Guys Hospital Research Ethics Committee approved the collection of families from the Newcomen Centre to identify families from the South East of England with specific language disorder. Ref No. 96/7/11. Cambridge Local Research Ethics Committee approved the CLASP project “Genome Search for susceptibility loci to language disorders” Ref No. LREC96/212. Ethical approval for the Manchester Language Study was given by the University of Manchester Committee on the Ethics of Research on Human Beings. Ref No. 03061 The Lothian Research Ethics Committee approved the project “Genetics of specific language impairment in children in Scotland” for the use of the Edinburgh samples. Ref. No. LREC/1999/6/20. The ethics department of the University of Chile approved the project “Genetic analysis of language-impaired individuals from the Robinson Crusoe Island”. Project Number 001-2010. Informed consent was given by all participants and/or, where applicable, their parents.

### Ascertainment of the Robinson Crusoe population

The Robinson Crusoe cohort was ascertained on the basis of phenotypic data from 61 children, between the ages of 3 years and 8 years, 11 months (i.e. the child cohort, described below) all of whom were descendants of the founder families and represents an extended cohort (including children who have turned 3 years of age since 2008) of that described in [33]. First-degree relatives of founder-related children found to meet criteria for SLI or typical language development were then also assessed for language performance (i.e. the family cohort, described below). Age constraints of available standardized tests meant that different language batteries were employed within the child and family cohorts.

### Phenotyping and selection of the Robinson Crusoe child cohort

The language ability of 61 children, all of whom were related to a founder individual, was assessed by tests of expressive and receptive language (Toronto Spanish Grammar Exploratory test, TEGE [58]) and phonology (Phonological simplification test (Test para Evaluar Procesos de Simplificación Fonológica—TEPROSIF [59]). Nonverbal IQ was tested using the Colombia Mental Maturity Scale [60]. In addition, all children were subjected to an auditory screen and oral motor exam [61]. All tests were validated and normalized in Chilean populations. On the basis of these tests, all children were classified into one of the three following categories:

1. “Specific Language Impairment)” (N = 16, 7 male, 9 female, 26.2%) defined as (i) performance >2SD below expected on TEPROSIF (for children aged 6 years or less) or performance >2 years below expected for chronological age on TEPROSIF (for children aged over 6 years) and/or performance below the 10^th^ percentile on either the receptive or expressive scales of the TEGE, (ii) nonverbal IQ not below the 10^th^ percentile, (iii) normal hearing, oral motor skills and neurological development.
2. “Typical language development” (N = 23, 8 male, 15 female, 37.7%) defined as (i) performance not >2SD below expected on TEPROSIF or performance >2 years below expected for chronological age on TEPROSIF (for children aged over 6 years) and performance above the 10^th^ percentile on both the receptive and expressive scales of the TEGE.
3. “Nonspecific language impairment” (N = 22, 13 male, 9 female, 36.1%) defined as (i) performance >2SD below expected on TEPROSIF or performance >2 years below expected for chronological age on TEPROSIF (for children aged over 6 years) and/or performance below the 10^th^ percentile on either the receptive or expressive scales of the TEGE, and (ii) nonverbal IQ >1SD below age-expected, and/or (iii) evidence of hearing loss or oral motor disability (e.g cleft lip) or abnormal neurological development.

The observed language deficits in the individuals diagnosed with SLI were typical of those described in other SLI cohorts and involved varied deficits across grammatical, morphosyntactical and receptive aspects of language, but not dialectic variations in intonation, vocabulary or phonology.

### Phenotyping and selection of the Robinson Crusoe family cohort

Since we were particularly interested in genetic contributions to SLI, our family cohort consisted of the first-degree relatives of the 39 founder-related children presenting with SLI or typical language development. All available first-degree family members (92 parents and siblings, 47 male, 45 female) were assessed for language difficulties using tests of verbal fluency (Barcelona test [62]) and verbal comprehension (Token test [63]). These family members included 11 parents who were not related to a founder member of the Island (referred to as non-founder-related parents). In addition to these formal language assessments, all individuals (or their parents or spouses) completed a family history interview (provided by P Tallal) [64], which specifically asks questions regarding language difficulties. On the basis of these data individuals were classified as either:

1. “Language-impaired” (N = 34, 15 male, 19 female, 37.0%, including 4 non-founder-related parents) if they scored below the 10^th^ percentile on either the Barcelona test or the token test or they self-reported a need for writing or reading support at school or a history of language support in the family history questionnaire.
2. “Typical language ability” (N = 58, 32 male, 26 female, 63.0%, including 7 non-founder-related parents) if they scored above the 10^th^ percentile on both the Barcelona test and the token test and they indicated no requirement for writing, reading or language support in the family history questionnaire.

### Exome sequencing of selected Robinson Crusoe children

Five Islanders (3 male, 2 female) from the child cohort who had been diagnosed with SLI were selected for exome sequencing. The selection of individuals for sequencing was based upon the amount and quality of DNA available, the severity of observed language impairment and their known relationships with other affected individuals. The five children were selected to cover the different branches of the founder pedigree and were descendants of the founder families (Fig. 1).

Exome capture was performed using 10μg of genomic DNA with a first generation (v1) Agilent SureSelect human exome kit (Agilent, Santa Clara, CA, USA), which provide an average target coverage of 80% of the exome at 56-fold across all samples. Sequencing of the generated fragments was performed on the SOLiD 4 sequencer (Life Technologies, Carlsbad, CA, USA). Color space reads were mapped to the human reference genome (hg18) in the SOLiD bioscope software (v1.2), which applies an iterative mapping approach. Variants were called using a diBayes algorithm [65] using high stringency settings, requiring calls on each strand. Small insertions and deletions were detected using the SOLiD Small Indel Tool. We assumed a binomial distribution with a probability of 0.5 of sequencing the variant allele at a heterozygous position. Given such a distribution, a minimum of ten reads would be required to provide a 99% probability that two or more reads contain an allele variant call. We filtered variant calls to have at least four unique (i.e. different start sites) variant reads with the variant being present in at least 15% of all reads.

To test the hypothesis that the founder brothers carried a rare causative genetic mutation, for our downstream analyses, we focused upon novel variants that were potentially deleterious. Each exome file was individually filtered to exclude nongenic, intronic (other than canonical splice sites) and synonymous variants. The remaining nonsynonymous and splice-site mutations were further filtered to exclude known sites of variation (as described in dbSNP, (build 130), publically available genome sequences and an in-house sequencing database). The remaining variants were then compared across exome samples to allow the selection of variants that occurred in 3 or more of the 5 children sequenced. A flow diagram of the methodology can be found in S1 Fig.. Shared novel, potentially deleterious variants discovered in the exome data were verified by Sanger sequencing. Primers for Sanger sequencing were designed in primer3 [66]. Primer sequences are available on request.

### Association analyses of selected variants in the Robinson Crusoe population

All novel nonsynonymous or canonical splice-site variants found to occur in 3 or more of the 5 exome samples were also genotyped in the wider child and family cohorts from the Robinson Crusoe population. We were able to obtain DNA samples for 35 founder-related children (from the SLI and typical language development child groups described above) and their family members (from the family cohort described above). Forty nine of these individuals (16 children, 22 parents (4 of whom were non-founder-related), 7 siblings and 4 half-siblings) were language impaired and 62 (19 children, 32 parents (7 of whom were non-founder-related), 9 siblings and 2 half-siblings) had language ability in the normal range. These families included the five children used in the exome sequencing. DNA was extracted from EDTA whole blood samples using a standard chloroform extraction protocol. All novel nonsynonymous or canonical splice-site variants identified from the exome screen were sequenced using a standard Sanger protocol in these 111 individuals.

The resultant genotype data were used to perform a family-based test of association within the MQLS-XM package [36,67]. This algorithm calculates a quasi-likelihood score which corrects the Chi-square statistic for relationships between individuals, providing accurate type I error rates [68]. The MQLS-XM extension allows for the accurate application of this statistic to X-linked markers [67]. The MQLS algorithm distinguishes between unaffected controls and controls of unknown phenotype, can incorporate phenotypic data from individuals who have not been genotyped [36] and is robust to the mis-specification of prevalence. It allows for the presence of both linkage and association effects in the test statistic and is computationally straightforward making it particularly suitable for large complex pedigrees in which cases and controls may be inter-related, as is the case in this study [36].

A full pedigree structure was generated that accounted for all known relationships between 111 individuals from the child and family cohorts and the two identified, shared, founder brothers. This pedigree included 288 individuals (141 males, 144 females and 85 founders (i.e. individuals with no parental information available—both original founders and incoming), 203 non-founders) over 5 generations. As described above, 111 individuals (including 11 non-founder-related parents) had full genotype and phenotype data, 11 individuals were also included who had phenotype data but no genotype data and the remaining 166 individuals had no phenotype or genotype data but defined relationships between the 111 genotyped individuals and the founder brothers. In the MQLS-XM analyses, the expected prevalence of SLI was set at 0.25 for males and 0.27 for females. These figures were derived from the child cohort described above.

Any variant that was significantly associated with language impairment in the population cohort was genotyped in 127 independent European population controls (ECACC, HRC-1 DNA Panel),441 independent South American controls; 320 individuals of Colombian descent and 121 individuals of Chilean origin. The Colombian controls were collected as part of a genetic demography study in the Colombian population, where all participants had to have four grandparents of local origin (provided by Luis Carvajal-Carmona and Maria Magdalena Echeverry). The Chilean controls were ascertained from the Santiago area and consisted of DNA from 30 male Chilean students (provided by P Villanueva) and from 91 female adult controls from a breast cancer study (provided by L Jara, University of Chile). Genome-wide SNP data indicated that these samples were of Amerindian and European ancestry. Note that both the European and South American control populations were unselected and, as such, were not screened for language ability.

### Linkage analysis of chromosome 4

Genome-wide linkage data for the Robinson Crusoe validation cohort have previously been reported [35]. These previous analyses included 6,090 SNPs and reported suggestive linkage (P<7.3×10^−4^) between SLI and chromosomes 2, 6, 7, 8, 9, 12, 13 and 17. In the current study, we had access to a new set of denser genotypes from the Robinson Crusoe population, generated with the Affymetrix Axiom GW-LAT 1 array (Affymetrix Inc, Santa Clara, CA, www.affymetrix.com), supplemented with a custom array designed to cover South American-specific variants which together included 1,141,741 SNPs.

929 SNPs across chromosome region chr4:46,000,000–49,000,000 (hg19) were selected to cover the chromosome region surrounding the *NFXL1* gene (reported transcript—chr4:47,849,258–47,916,680, hg19). SNP data were filtered within PLINK [69] to remove markers in close linkage disequilibrium (r^2^>0.5) resulting in a linkage dataset of 54 independent SNPs that were appended with rs144169475 genotype data and analysed for linkage in MERLIN [70]. Linkage disequilibrium between these SNPs and rs144169475 are provided in S3 Fig.

Since linkage packages were unable to analyse genome-wide data for the 321-bit Robinson Crusoe validation pedigree as a whole, it was broken into sub-pedigrees manually selected on the basis of closest shared ancestor. We employed linkage sub-pedigrees and linkage methods analogous to those described in the previous linage study [35]; Seven extended families of 20–24 bits (where a bit is defined as twice the number of non-founders—the number of founders) were analysed for linkage under parametric and nonparametric models with MERLIN (S2 Fig.) Parametric linkage analyses were performed under a model which reflected the observed nature of rs144169475 (assuming a disease frequency of 26.2% (as observed in the Robinson Crusoe children) and penetrance of 0.76 (as observed in the Robinson Crusoe validation cohort). Nonparametric linkage results are reported as P-values derived from the Kong and Cox exponential model, which can be more powerful in large pedigrees [71]. Expected allele frequencies were derived from the 1000 Genomes AMR super-population (integrated phase 1, accessed March 2014) which includes 181 independent South American individuals (60 Colombians from Medellin, Colombia (CLM), 66 individuals with Mexican ancestry in Los Angeles (MXL) and 55 Puerto Ricans from Puerto Rico (PUR)) [40].

### Functional effects of identified variants

Putative functional effects of associated variants were evaluated using MutationTaster [72]. MutationTaster uses a Bayes classifier which integrates information from various biomedical databases and analysis tools to evaluate the possible pathogenicity of coding variants. MutationTaster considers evolutionary conservation at both the nucleotide and amino acid level, splice-site changes, loss of protein motifs or features and changes that might affect the level of mRNA expression and stability within a single tool to classify variants as a “disease mutation” or a “polymorphism”. A p-value is given to indicate “the security” of the prediction [72]. The MutationTaster algorithm was trained using more than 390,000 known disease mutations from HGMD and more than 6,800,000 SNPs and Indel polymorphisms from the 1000 Genomes Project.

For each of the variants highlighted, we also present the SIFT and polyphen-2 scores. In contrast to MutationTaster, the SIFT and PolyPhen algorithms primarily consider protein sequences, motifs and structures to assign pathogenicity and therefore can only be applied to coding changes. SIFT performs a multiple alignment of closely related protein sequences to identify conserved motifs and assign a probability that a given amino acid substitution is pathogenic [73]. PolyPhen-2 uses a Bayes classifier to consider the property of the reference and variant amino acids, the amino acid conservation, protein motifs and 3D protein structure to derive a probability that a mutation is damaging [74]. SIFT scores vary between 0 and 1. Amino acid substitutions are classified as “deleterious” for scores ≤0.05 and “tolerated” for scores >0.05. In Polyphen-2, two training models are available—HumDiv, which is more appropriate for the identification of fully penetrant Mendelian mutations and HumVar, which is more appropriate for the classification of rare alleles at loci potentially involved in complex phenotypes. PolyPhen scores from both of these models vary from 0 to 1, where 0 represents a variant with no functional effect. Functional effects are classified as “benign”, “possibly damaging”, or “probably damaging”, depending on whether the posterior probability falls above or below the appropriate false positive thresholds.

### Sequencing of candidate genes in SLIC cohort

In order to further investigate the role of NFXL1 variants in SLI, the coding regions of the *NFXL1* gene were subsequently sequenced in 117 unrelated British children affected by SLI. These children formed part of the SLI Consortium (SLIC) collection, which has previously been described in detail [7,37,39]. In short, the probands were collected from four sites across the UK (The Newcomen Centre at Guy’s Hospital, London, the Cambridge Language and Speech Project (CLASP) [75], the Child Life and Health Department at the University of Edinburgh [76] and the Manchester Language Study [77]). All probands were selected to have receptive and/or expressive language skills (as assessed by the Clinical Evaluation of Language Fundamentals (CELF-IV-R) [78]) more than 1.5SD below the normative mean for his or her age and non-verbal IQ (as measured by the Wechsler Intelligence Scale for Children [79]) in the “normal” range (>80).

The concentration of genomic DNA samples from 117 independent SLIC probands was quantified by picogreen and each sample normalized to 10ng/μl. Individual DNAs were pooled prior to PCR amplification. Following PCR, the amplicons were fragmented, end-repaired and adapter-ligated. The prepared and tagged libraries were then multiplexed before paired-end sequencing in a single lane of flow-cell on an Illumina HiSeq 2000 (Illumina Inc, SanDiego, CA, www.illumina.com). Sequences were aligned against human reference sequence (37d5) using STAMPY [80] and variants called by the Syzygy (1.2.6) algorithm to create a VCF file. Syzygy implements a Bayes likelihood calculation to allow a base calling strategy that is particularly suited to the calling of variants in pooled samples, in which the frequency of reads containing a rare variant will be lower than expected [81]. Identified sequence variants were annotated within the SNPeff package allowing the identification of coding variants [82]. Individual DNAs from all pools that contained a nonsynonymous coding variant with an expected frequency of <5% were resequenced by Sanger sequencing using primers designed with the primer3 software [66]. This allowed the verification of the variants, the derivation of true variant frequencies across pools and the identification of the individuals who carried the variant.

The allele frequencies of coding variants discovered in SLIC probands were compared to those observed in 4679 individuals of European ancestry across publically available control databases; the 1000 genomes project (the European (EUR) super-population from integrated phase 1, accessed March 2014) [40] which includes 379 independent European individuals (89 British in England and Scotland, 93 Finnish in Finland, 14 Iberian populations in Spain, 98 Toscani in Italy and 85 Utah residents with Northern and Western European ancestry) and the European American (EA) cohort from the exome variant server (ESP6500 SI-V2, accessed March 2014) (http://evs.gs.washington.edu/EVS/) which includes data from 4300 independent individuals of European American ancestry. The 1000 genomes samples are unselected controls while the EVS samples are selected to include controls, extremes of specific traits (LDL and blood pressure) and specific diseases (early onset myocardial infarction and early onset stroke). Allele frequencies were compared between SLIC probands and controls using a two-tailed Fisher’s exact test with 1 degree of freedom. Calculations were performed in the graphpad online calculator (http://www.graphpad.com/). Where given variants were observed in alternative populations, these data are reported but were not included in the statistical analyses since population admixture and stratification can lead to false positives, especially when investigating rare variants [83].

## Supporting Information

S1 Fig A flow diagram showing the filtering of the exome data. Blue boxes show each filter step and red boxes describe exclusion criteria involved in each step
(PDF)

S2 Fig Structure of pedigrees used for linkage analyses (redrawn using data from [35])). Seven pedigrees of no more than 24-bits were used for linkage analyses. Individuals with language impairment are colored in black. Individuals with typical language are denoted in white. Individuals with unknown phenotype are shaded grey.
(PDF)

S3 Fig Linkage disequilibrium between markers across *NFXL1* region. a—Linkage disequilibrium between all genotyped markers (n = 929) across chr4:46–49Mb (hg19). b—Linkage disequilibrium between all analyzed markers (n = 55) across chr4:46–49Mb (hg19), after pruning for r^2^>0.5. Position of *NFXL1* gene is shown by red box. Plots were generated in haploview (http://www.broadinstitute.org/scientific-community/science/programs/medical-and-population-genetics/haploview/haploview) using linkage pedigrees (as shown in S2 Fig.). Color scheme is standard haploview colour scheme (blue—D’ = 1, LOD<2; white—D’<1, LOD<2; pink/red—LOD≥2).
(PDF)

S4 Fig Linkage across the *NFXL1* region. No linkage was observed to the NFXL1 region of chromosome 4 under parametric and non-parametric models using a dense SNP array. The approximate position of the *NFXL1* gene is indicated by the red box on the X axis.
(PDF)

S1 Table Exome sequencing metrics. (PDF)

S2 Table Regions of suggestive linkage in the Robinson Crusoe population (as presented in [35]). (PDF)

S3 Table All variants found under the peaks of previous linkage (as reported in [35]) that were shared across all 5 exome samples. (PDF)

S4 Table Genotype reconstruction simulations. The *NFXL1* variant has an expected population frequency of between 0.033 (1000 genomes CLM) and 0.09 (1000 genomes PUR) and is predicted to be present in both founder brothers (frequency in founder brothers of 0.5). Given the population structure, it would therefore be expected to be present in the current population at a frequency of between 0.08 (MAF = 0.03) and 0.14 (MAF = 0.10). Although the frequency of the *NFXL1* variant in the founder-related individuals of the Robinson Crusoe validation cohort was at this expected level (0.125), the variant allele was found to cosegregate with language impairment; the frequency of the *NFXL1* variant in the founder-related individuals with SLI was above expected (0.194) while that of founder-related individuals with typical language was below expected (0.048), supporting a pathogenic role for this allele.
(PDF)

S1 Text Genotype reconstruction simulations. (PDF)
