# Correction: Exome Sequencing in an Admixed Isolated Population IndicatesNFXL1 Variants Confer a Risk for Specific Language Impairment

**DOI:** 10.1371/journal.pgen.1005336

**Published:** 2015-06-26

**Authors:** Pía Villanueva, Ron Nudel, Alexander Hoischen, María Angélica Fernández, Nuala H. Simpson, Christian Gilissen, Rose H. Reader, Lillian Jara, María Magdalena Echeverry, Clyde Francks, Gillian Baird, Gina Conti-Ramsden, Anne O’Hare, Patrick F. Bolton, Elizabeth R. Hennessy, SLI Consortium, Hernán Palomino, Luis Carvajal-Carmona, Joris A. Veltman, Jean-Baptiste Cazier, Zulema De Barbieri, Simon E. Fisher, Dianne F. Newbury

The name of the ninth author is incorrect. Maria Magdalena Echeverry should read María Magdalena Echeverry.

The affiliation for Pía Villanueva is incorrect. The affiliation should read Department of Speech, Language and Hearing Sciences, Faculty of Medicine, University of Chile, Santiago, Chile.

The affiliation for Anne O’Hare is incorrect. The affiliation should read Child Life & Health, School of Clinical Sciences, University of Edinburgh, Edinburgh, United Kingdom.

There is an error in the first paragraph of page 4. The sentence: "The contribution of identified risk variants is subsequently validated by performing targeted sequencing of candidate genes in a UK-based cohort of individuals affected by SLI," should read: "The contribution of identified risk variants is subsequently validated by performing targeted sequencing of a candidate gene in a UK-based cohort of individuals affected by SLI."

There are errors in the Author Contributions. The correct contributions are:

Conceived and designed the experiments: PV RN AH MAF CG LJ CF GB GCR AOH PFB ERH SLIC HP LCC JAV JBC ZDB SEF DFN Performed the Chilean fieldwork: PV MAF LJ ZDB Performed the genetic experiments: RN AH NHS CG RHR LCC JAV JBC SEF DFN Analyzed the phenotypic data: PV MAF LJ HP ZDB DFN Analyzed the genetic data: RN AH NHS CG RHR LCC JBC SEF DFN Contributed reagents/materials/analysis tools: PV AH MAF CG LJ MME GB GCR AOH PFB ERH SLIC LCC JAV ZDB SEF DFN Wrote the manuscript: PV RN JBC SEF DFN
