# Supplementary material for: Exome Sequencing in an Admixed Isolated Population Indicates NFXL1 Variants Confer a Risk for Specific Language Impairment
Source: PLoS Genet. 2015 Mar 17;11(3):e1004925. doi: 10.1371/journal.pgen.1004925 (PMC4363375; doi:10.1371/journal.pgen.1004925)
Supplement: S1 Fig — Blue boxes show each filter step and red boxes describe exclusion criteria involved in each step (PDF) [file pgen.1004925.s001.pdf]

Figure S1 – A flow diagram showing the filtering of the exome data.

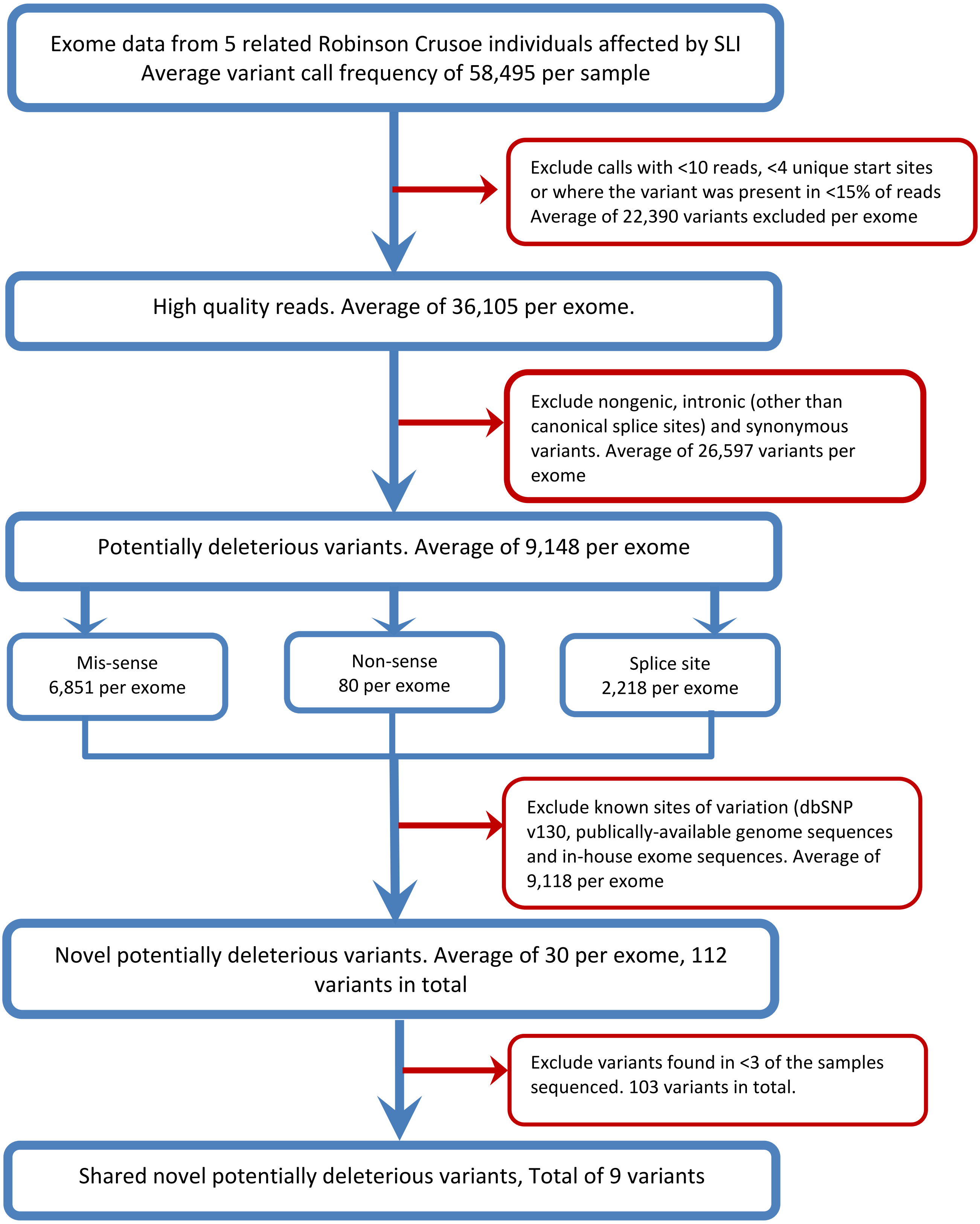

Blue boxes show each filter step and red boxes describe exclusion criteria involved in each step
