# Supplementary material for: Exome Sequencing in an Admixed Isolated Population Indicates NFXL1 Variants Confer a Risk for Specific Language Impairment
Source: PLoS Genet. 2015 Mar 17;11(3):e1004925. doi: 10.1371/journal.pgen.1004925 (PMC4363375; doi:10.1371/journal.pgen.1004925)
Supplement: S2 Fig — Seven pedigrees of no more than 24-bits were used for linkage analyses. Individuals with language impairment are colored in black. Individuals with typical language are denoted in white. Individuals with unknown phenotype are shaded grey. (PDF) [file pgen.1004925.s002.pdf]

Figure S2 - Structure of pedigrees used for linkage analyses (redrawn using data from [35])).

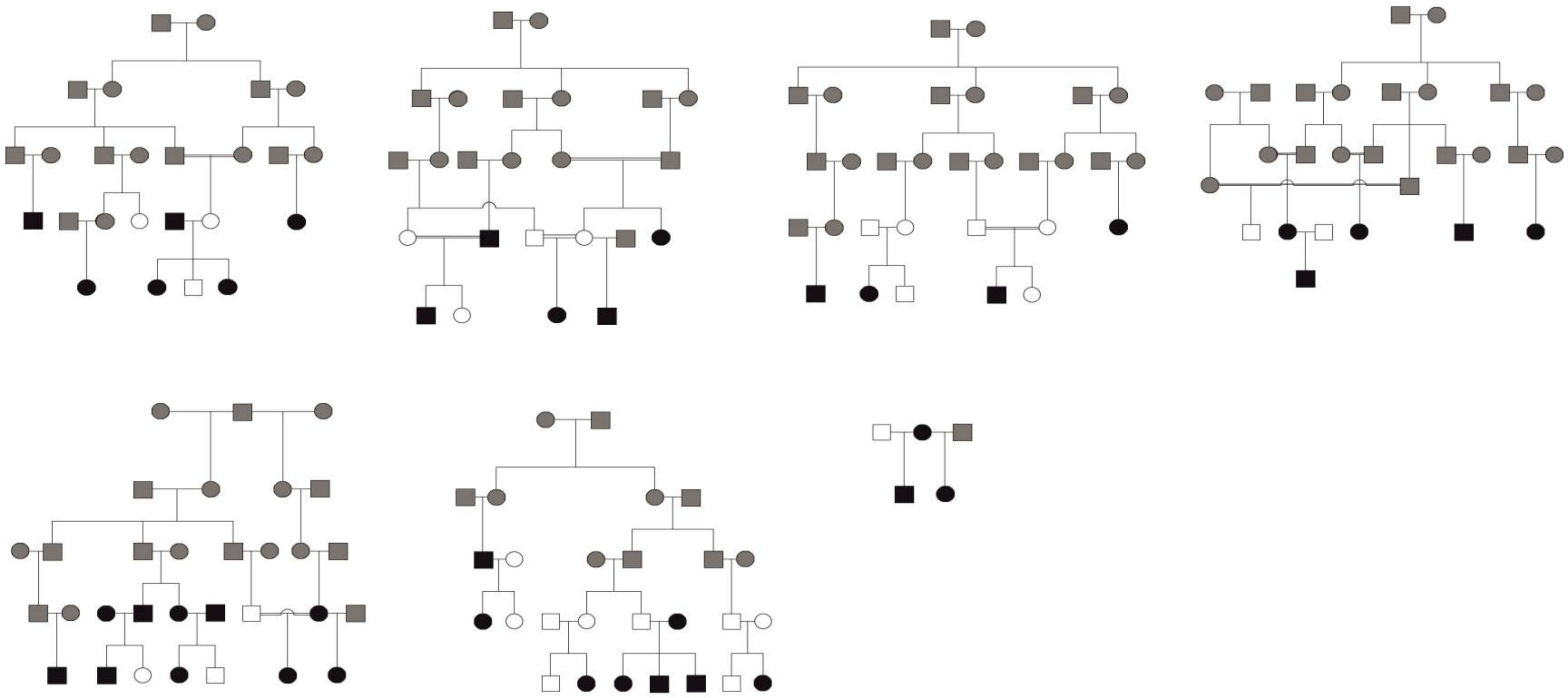

Seven pedigrees of no more than 24-bits were used for linkage analyses.

Individuals with language impairment are colored in black. Individuals with typical language are denoted in white. Individuals with unknown phenotype are shaded grey.
