# Supplementary material for: Exome Sequencing in an Admixed Isolated Population Indicates NFXL1 Variants Confer a Risk for Specific Language Impairment
Source: PLoS Genet. 2015 Mar 17;11(3):e1004925. doi: 10.1371/journal.pgen.1004925 (PMC4363375; doi:10.1371/journal.pgen.1004925)
Supplement: S3 Fig — a—Linkage disequilibrium between all genotyped markers (n = 929) across chr4:46–49Mb (hg19). b—Linkage disequilibrium between all analyzed markers (n = 55) across chr4:46–49Mb (hg19), after pruning for r2>0.5. Position of NFXL1 gene is shown by red box. Plots were generated in haploview (http://www.broadinstitute.org/scientific-community/science/programs/medical-and-population-genetics/haploview/haploview) using linkage pedigrees (as shown in S2 Fig.). Color scheme is standard haploview colour scheme (blue—D’ = 1, LOD<2; white—D’<1, LOD<2; pink/red—LOD≥2). (PDF) [file pgen.1004925.s003.pdf]

Figure S3 - Linkage disequilibrium between markers across *NFXL1* region.

a.

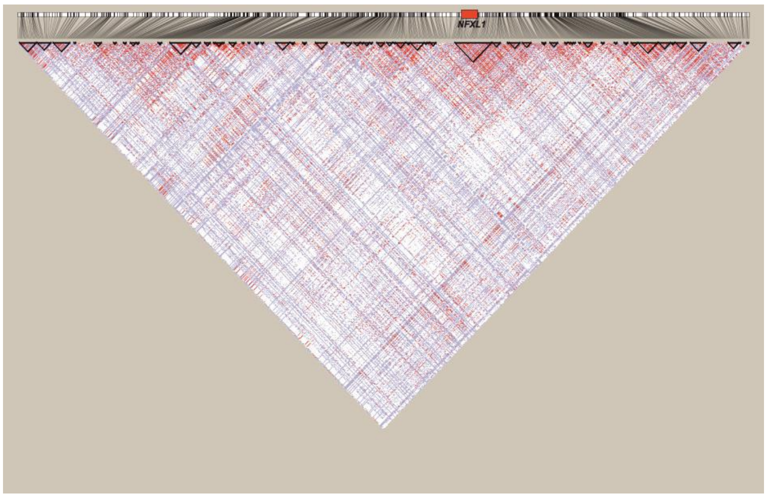

b.

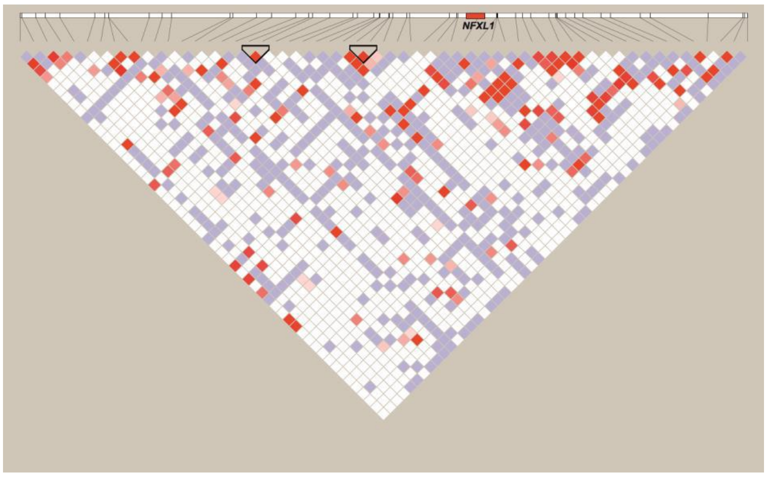

a - Linkage disequilibrium between all genotyped markers (n=929) across chr4:46-49Mb (hg19)

b - Linkage disequilibrium between all analyzed markers (n=55) across chr4:46-49Mb (hg19), after pruning for r^2>0.5

Position of *NFXL1* gene is shown by red box. Plots were generated in haploview (http://www.broadinstitute.org/scientific-community/science/programs/medical-and-population-genetics/haploview/haploview) using linkage pedigrees (as shown in Figure S2).

Color scheme is standard haploview colour scheme (blue – D'=1, LOD<2; white - D'<1, LOD<2; pink/red – LOD≥2).
