# Supplementary material for: Exome Sequencing in an Admixed Isolated Population Indicates NFXL1 Variants Confer a Risk for Specific Language Impairment
Source: PLoS Genet. 2015 Mar 17;11(3):e1004925. doi: 10.1371/journal.pgen.1004925 (PMC4363375; doi:10.1371/journal.pgen.1004925)
Supplement: S4 Fig — No linkage was observed to the NFXL1 region of chromosome 4 under parametric and non-parametric models using a dense SNP array. The approximate position of the NFXL1 gene is indicated by the red box on the X axis. (PDF) [file pgen.1004925.s004.pdf]

Figure S4 – Linkage across the *NFXL1* region

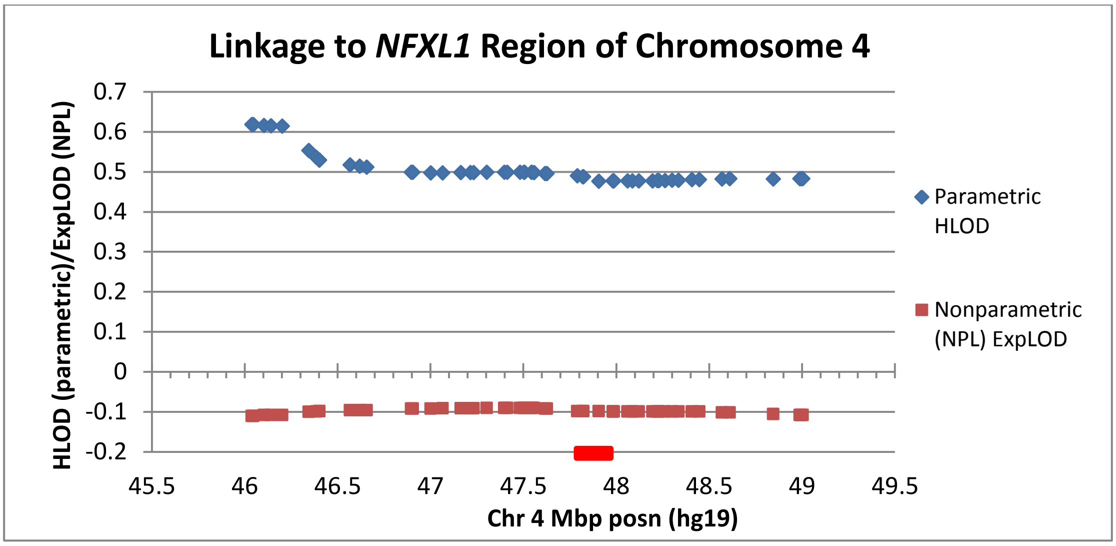

No linkage was observed to the NFXL1 region of chromosome 4 under parametric and non-parametric models using a dense SNP array. The approximate position of the *NFXL1* gene is indicated by the red box on the X axis.
