# Supplementary material for: Exome Sequencing in an Admixed Isolated Population Indicates NFXL1 Variants Confer a Risk for Specific Language Impairment
Source: PLoS Genet. 2015 Mar 17;11(3):e1004925. doi: 10.1371/journal.pgen.1004925 (PMC4363375; doi:10.1371/journal.pgen.1004925)
Supplement: S1 Table — (PDF) [file pgen.1004925.s005.pdf]

Table S1 – Exome sequencing metrics

	SLI3	SLI8	SLI9	SLI12	SLI15	Average across 5 sequence samples	All sequence samples combined
Min depth	2	2	1	2	2	2	1
Max depth	2,142	1,234	2,843	1,602	3,721	2,308	3,721
Average depth	12	24	39	19	35	26	34
Median depth	9	21	22	18	20	18	17
% variants with ≥5x coverage	63.7%	87.3%	88.3%	85.2%	88.4%	82.6%	82.2%
% variants with ≥10x coverage	47.5%	71.9%	73.8%	68.7%	72.3%	66.8%	66.5%
% variants with ≥20x coverage	29.6%	52.5%	53.9%	47.4%	50.6%	46.8%	46.6%
Variants called	63,031	59,822	58,769	52,881	57,972	58,495	145,369
Variants that map to genes	43,930	50,112	49,543	43,075	49,721	47,276	111,491
Variants in exons	15,200	19,837	17,326	16,419	18,242	17,405	38,773
Variants in splice sites	2,645	3,304	3,143	2,879	3,042	3,003	6,516
Nonsense variants	75	137	72	90	157	106	301
Missense variants	7,258	9,629	8,089	7,805	9,114	8,379	19,548
Deletions	766	1,266	1,478	989	1,183	1,136	2,942
Insertions	471	798	912	648	734	713	1,567
