# Supplementary material for: Exome Sequencing in an Admixed Isolated Population Indicates NFXL1 Variants Confer a Risk for Specific Language Impairment
Source: PLoS Genet. 2015 Mar 17;11(3):e1004925. doi: 10.1371/journal.pgen.1004925 (PMC4363375; doi:10.1371/journal.pgen.1004925)
Supplement: S2 Table — (PDF) [file pgen.1004925.s006.pdf]

Table S2 - Regions of suggestive linkage in the Robinson Crusoe population (as presented in [35]).

Linkagepeak (hg19)	Linkagepeak (hg18)	Start SNP	End SNP	Start bp (hg19)	End bp (hg19)	max NPL score (p-value)	SNP (chr posn, hg19) of max CEPH NPL score
chr2:8038605-9916389	chr2:7956056-9833840	rs1364054	rs12995394	8,038,605	9,916,389	3.9 (5.0x10-5)	rs3102960 (8312367)
chr6:8525127-10528561	chr6:8470126-10636547	rs1410766	rs560194	8,525,127	10,528,561	3.96 (4.0x10-5)	rs761116 (9458096)
chr6:159048542-162847872	chr6:158968530-162767862	rs182429	rs1333962	159,048,542	162,847,872	7.56 (3.2x10-14)	rs927450 (159761064) -rs675162 (160355293)
chr7:112475603-159225515	chr7:112262839-158821424	rs7817	rs455030	112,475,603	159,225,515	6.73 (4.0x10-11)	rs1524341 (147009597) - rs1024676 (147018769)
chr8:70571274-72653781	chr8:70733828-72816335	rs268564	rs1440369	70,571,274	72,653,781	3.95 (4.0x10-5)	rs268564 (70571274)
chr8:139553440-144841669	chr8:139622622-144913657	rs741465	rs1735169	139,553,440	144,841,669	3.69 (0.0001)	rs1375062 (140955682) - rs768803 (142124264)
chr9:602986-21732913	chr9:592986-21722913	rs1532310	rs1414237	602,986	21,732,913	3.72 (0.0001)	rs717081 (20287141)
chr12:132922352-133201580	chr12:131432425-131711653	rs1278602	rs7960480	132,922,352	133,201,580	6.14 ((9.9x10-10)	rs595241 (133077993) - rs632610 (133084132)
chr13:34588741-40958916	chr13:33486741-39856916	rs12429306	rs7799	34,588,741	40,958,916	4.78 (8.0x10-7)	rs980285 (38465795)
chr13:83943347-94082682	chr13:82841348-92880683	rs1334857	rs719223	83,943,347	94,082,682	3.49 (0.0002)	rs996297 (85433362) - rs979969 (87644846)
chr17:80188016-82928872	chr17:75776410-78480037	rs3829611	rs3785513	80,188,016	82,928,872	4.49 (3.0x10-6)	rs1046875 (82727550) - rs1046896 (82727657)
