# Supplementary material for: Exome Sequencing in an Admixed Isolated Population Indicates NFXL1 Variants Confer a Risk for Specific Language Impairment
Source: PLoS Genet. 2015 Mar 17;11(3):e1004925. doi: 10.1371/journal.pgen.1004925 (PMC4363375; doi:10.1371/journal.pgen.1004925)
Supplement: S3 Table — (PDF) [file pgen.1004925.s007.pdf]

Table S3 - All variants found under the peaks of previous linkage (as reported in [35]) that were shared across all 5 exome samples.

Position (hg19)	Ref	Variant	Variation Type	SNP id	Variant freq (dbSNP138)	Gene name	Gene id	Gene component	AminoAcid changes	mRNA changes	phyloP	SIFT score	Grantham Score	Notes
chr2:9419411-9419411	G	A	substitution	rs10193043	0.706284	*ASAP2*	NM_001135191	INTRON_REGION			-1.23			known common (>5%) SNP, intronic
chr6:10214600-10214600	G	A	substitution	rs9366321	0.849656						-0.38			known common (>5%) SNP, non-genic
chr6:160198395-160198395	G	A	substitution	rs3465	0.281313	*ACAT2*	NM_005891	EXON_REGION	none	819G>A	0.40			known common (>5%) SNP, nonsynonymous
chr6:160202167-160202167	G	A	substitution	rs2295899	0.418335	*TCP1*	NM_001008897	INTRON_REGION			-0.39			known common (>5%) SNP, intronic
chr6:160211636-160211636	G	A	substitution	rs1128670	0.543881	*MRPL18*	NM_014161	EXON_REGION	R6Q	17G>A	3.46	0.34	43	known common (>5%) SNP
chr6:160234625-160234625	G	C	substitution	rs13220869	0.59001	*PNLDC1*	NM_173516	SA_SITE			-2.30			known common (>5%) SNP
chr6:160505199-160505199	C	G	substitution	rs614754	0.978161	*IGF2R*	NM_000876	EXON_REGION	none	6051C>G	-0.64			known common (>5%) SNP, nonsynonymous
chr6:160551204-160551204	G	C	substitution	rs683369	0.856156	*SLC22A1*	NM_153187	EXON_REGION	L160F	480G>C	-0.44	0.34	22	known common (>5%) SNP
chr6:160677614-160677614	G	C	substitution	rs2774230	0.254709	*SLC22A2*	NM_003058	INTRON_REGION			-0.93			known common (>5%) SNP, intronic
chr6:160888084-160888084		A	insertion	rs71033580	1	*LPAL2*	NR_028093	UTR			-4.23			known common (>5%) SNP, UTR
chr6:161139502-161139502	G	A	substitution	rs2295368	0.371684	*PLG*	NM_000301	INTRON_REGION			-1.27			known common (>5%) SNP, intronic
chr6:161469774-161469774	G	A	substitution	rs4559074	0.974063	*MAP3K4*	NM_006724	EXON_REGION	R157H	470G>A	2.57	0.27	29	known common (>5%) SNP
chr6:161531006-161531006	G	C	substitution	rs4709493	0.930402	*MAP3K4*	NM_005922	INTRON_REGION			0.35			known common (>5%) SNP, intronic
chr7:113518502-113518502	A	T	substitution	rs2974938	0.96805	*PPP1R3A*	NM_002711	EXON_REGION	L882H	2645A>T	-1.04	0	99	known common (>5%) SNP
chr7:113519719-113519719	A	T	substitution	rs2974944	0.996537	*PPP1R3A*	NM_002711	EXON_REGION	N476K	1428A>T	0.42	0.91	94	known common (>5%) SNP
chr7:120428799-120428799	C	A	substitution	rs41623	0.19992	*TSPAN12*	NM_012338	EXON_REGION	none	765C>A	-0.66			known common (>5%) SNP, nonsynonymous
chr7:120450658-120450658	G	A	substitution	rs7805733	0.829338	*TSPAN12*	NM_012338	INTRON_REGION			0.80			known common (>5%) SNP, intronic
chr7:120764477-120764477	G	C	substitution	rs1524498	0.43903	*C7orf58*	NM_001105533	EXON_REGION	none	1011G>C	0.42			known common (>5%) SNP, nonsynonymous
chr7:121059871-121059871	G	A	substitution	rs2707500	0.680917						0.35			known common (>5%) SNP, non-genic
chr7:123269118-123269118	G	C	substitution	rs4731112	0.744237	*ASB15*	NM_080928	EXON_REGION	G357A	1070G>C	4.02	1	60	known common (>5%) SNP
chr7:124387092-124387092	C	G	substitution	rs3735270	0.410128	*GPR37*	NM_005302	EXON_REGION	none	1329C>G	0.44			known common (>5%) SNP, nonsynonymous
chr7:131815343-131815343	G	A	substitution	rs10954361	0.736126	*PLXNA4*	NM_020911	SA_SITE			0.87			known common (>5%) SNP
chr7:134849280-134849280	T	A	substitution	rs292501	0.913054	*TMEM140*	NM_018295	EXON_REGION	F29L	87T>A	0.64	1	22	known common (>5%) SNP
chr7:134873355-134873355	G	A	substitution	rs292564	0.720347	*WDR91*	NM_014149	INTRON_REGION			-1.86			known common (>5%) SNP, intronic
chr7:135304273-135304273	G	C	substitution	rs7810767	0.982239	*NUP205*	NM_015135	EXON_REGION	E1356Q	4066G>C	2.27		29	known common (>5%) SNP
chr7:138200117-138200117	A	T	substitution	rs3757381	0.763263	*TRIM24*	NM_003852	INTRON_REGION			0.33			known common (>5%) SNP, intronic
chr7:138418910-138418910	G	A	substitution	rs1026435	0.695556	*ATP6V0A4*	NM_130840	EXON_REGION	none	1662G>A	-1.17			known common (>5%) SNP
chr7:140082363-140082363	G	A	substitution	rs6968618	0.756045	*SLC37A3*	NM_207113	UTR			-1.65			known common (>5%) SNP, UTR
chr7:141478574-141478574	G	C	substitution	rs2234001	0.492189	*TAS2R4*	NM_016944	EXON_REGION	V96L	286G>C	1.01	1	32	known common (>5%) SNP
chr7:141478800-141478800	G	A	substitution	rs2234002	0.456955	*TAS2R4*	NM_016944	EXON_REGION	S171N	512G>A	-1.96	1	46	known common (>5%) SNP
chr7:141537945-141537945	T	A	substitution	rs11766230	0.049587	*PRSS37*	NM_001008270	INTRON_REGION			-0.51			known SNP, intronic
chr7:141673345-141673345	C	G	substitution	rs713598	0.471632	*TAS2R38*	NM_176817	EXON_REGION	A49P	145C>G	2.05	1	27	known common (>5%) SNP
chr7:143512184-143512184	G	A	substitution	rs72503048	1	*LOC154761*	NR_015421	UTR			-0.50			known common (>5%) SNP, UTR
chr7:146997203-146997203	T	A	substitution	rs2692185	0.246649	*CNTNAP2*	NM_014141	INTRON_REGION			0.49			known common (>5%) SNP, intronic
chr7:147964091-147964091	G	A	substitution	rs3801976	0.787494	*CNTNAP2*	NM_014141	INTRON_REGION			-2.08			known common (>5%) SNP, intronic
chr7:148106490-148106490	G	A	substitution	rs9648691	0.556152	*CNTNAP2*	NM_014141	EXON_REGION	none	3723G>A	-0.46			known common (>5%) SNP, nonsynonymous
chr7:148851213-148851213	G	A	substitution	rs917124	0.719752	*ZNF398*	NM_170686	EXON_REGION	none	201G>A	-0.80			known common (>5%) SNP, nonsynonymous
chr7:150731338-150731338	G	C	substitution	rs2303923	0.711312	*ABCB8*	NM_007188	INTRON_REGION			-0.73			known common (>5%) SNP, intronic
chr7:150773072-150773072	G	C	substitution	rs2303939	0.591346	*SLC4A2*	NM_003040	INTRON_REGION			-1.50			known common (>5%) SNP, intronic
chr7:151932819-151932819	T	A	substitution	rs80348298	0.5	*MLL3*	NM_170606	INTRON_REGION			1.39			known common (>5%) SNP, intronic
chr7:151932824-151932824	A	T	substitution	rs78860638	0.5	*MLL3*	NM_170606	INTRON_REGION			-0.48			known common (>5%) SNP, intronic
chr7:151932897-151932897	C	A	substitution	rs73728782	0.5	*MLL3*	NM_170606	SD_SITE			4.96			known common (>5%) SNP
chr7:151945101-151945101	G	C	substitution	rs3896406	1	*MLL3*	NM_170606	EXON_REGION	none	2418G>C	-0.84			known common (>5%) SNP, nonsynonymous
chr7:151962176-151962176	T	A	substitution	rs62478356	0	*MLL3*	NM_170606	EXON_REGION	none	1131T>A	0.63			known SNP, nonsynonymous
chr7:154862621-154862621	T	A	substitution	rs6320	0.273557	*HTR5A*	NM_024012	EXON_REGION	none	12T>A	-0.81			known common (>5%) SNP, nonsynonymous
chr7:157370688-157370688	C	G	substitution	rs122004	0.075527	*PTPRN2*	NM_130842	INTRON_REGION			-1.42			known common (>5%) SNP, intronic
chr7:158438186-158438186	G	A	substitution	rs2290393	0.503484	*NCAPG2*	NM_017760	INTRON_REGION			0.28			known common (>5%) SNP, intronic
chr7:158455089-158455089	G	A	substitution	rs4484634	0.933361	*NCAPG2*	NM_017760	INTRON_REGION			0.31			known common (>5%) SNP, intronic
chr8:139614436-139614436	G	A	substitution	rs4394401	0.875817	*COL22A1*	NM_152888	INTRON_REGION			-1.48			known common (>5%) SNP, intronic
chr8:139626176-139626176	C	G	substitution	rs7000261	0.123675	*COL22A1*	NM_152888	SA_SITE			0.99			known common (>5%) SNP
chr8:141034189-141034189	A	T	substitution	rs9792174	0.393066	*TRAPPC9*	NM_001160372	SA_SITE			0.59			known common (>5%) SNP
chr8:141559358-141559358	G	A	substitution	rs2292781	0.4134	*EIF2C2*	NM_001164623	EXON_REGION	none	1443G>A	-6.26			known common (>5%) SNP, nonsynonymous
chr8:141568622-141568622	G	A	substitution	rs2292778	0.628608	*EIF2C2*	NM_001164623	EXON_REGION	none	840G>A	-1.75			known common (>5%) SNP, nonsynonymous
chr8:142204326-142204326	C	G	substitution	rs1045248	0.550396	*DENND3*	NM_014957	EXON_REGION	none	3591C>G	-1.15			known common (>5%) SNP, nonsynonymous
chr8:142228909-142228909	G	A	substitution	rs753778	0.342997	*SLC45A4*	NM_001080431	EXON_REGION	P226L	677G>A	0.52	0	98	known common (>5%) SNP
chr8:144406881-144406881	G	A	substitution	rs12680498	0.452011	*TOP1MT*	NM_052963	INTRON_REGION			-2.95			known common (>5%) SNP, intronic
chr8:144801681-144801681	G	A	substitution	rs11778657	0.567132	*MAPK15*	NM_139021	INTRON_REGION			-0.69			known common (>5%) SNP, intronic

Position (hg19)	Ref	Variant	Variation Type	SNP id	Variant freq (dbSNP138)	Gene name	Gene id	Gene component	AminoAcid changes	mRNA changes	phyloP	SIFT score	Grantham Score	Notes
chr8:144808747-144808747	G	A	substitution	rs11136321	0.606372	*FAM83H*	NM_198488	EXON_REGION	none	2884G>A	1.12			known common (>5%) SNP, nonsynonymous
chr8:144811338-144811338	C	G	substitution	rs9969600	0.000284	*FAM83H*	NM_198488	EXON_REGION	Q201H	603C>G	0.86	0.02	24	known SNP, variant found in 98% of reads, SIFT low confidence
chr9:660290-660290	G	A	substitution	rs9407340	0.84253	*KANK1*	NM_015158	UTR			0.79			known common (>5%) SNP, UTR
chr9:967981-967981	G	C	substitution	rs279895	0.090763	*DMRT1*	NM_021951	SA_SITE			0.97			known common (>5%) SNP
chr9:2170512-2170512	G	C	substitution	rs3793511	0.44369	*SMARCA2*	NM_139045	INTRON_REGION			3.13			known common (>5%) SNP, intronic
chr9:4722685-4722685	C	G	substitution	rs10758645	0.478291	*AK3*	NM_016282	INTRON_REGION			-0.95			known common (>5%) SNP, intronic
chr9:5081780-5081780	G	A	substitution	rs2230724	0.613773	*JAK2*	NM_004972	EXON_REGION	none	2490G>A	-0.88			known common (>5%) SNP, nonsynonymous
chr9:12973305-12973305	C	G	substitution	rs1887883	0.00535						1.64			known SNP, non-genic
chr9:14921722-14921722	T	A	substitution	rs2030594	0.293915						0.61			known common (>5%) SNP, non-genic
chr9:15055607-15055607	G	A	substitution	rs4445325	0.408491						-0.58			known common (>5%) SNP, non-genic
chr9:15055921-15055921	C	G	substitution	rs7022876	0.620155						-1.20			known common (>5%) SNP, non-genic
chr9:15056034-15056034	G	C	substitution	rs4740598	0.757162						-0.35			known common (>5%) SNP, non-genic
chr9:15527569-15527569	G	A	substitution	rs28709032	0.542086						0.73			known common (>5%) SNP, non-genic
chr9:17409366-17409366	G	A	substitution	rs2780211	0.859498	*CNTLN*	NM_017738	EXON_REGION	none	2691G>A	1.01			known common (>5%) SNP, nonsynonymous
chr9:17464402-17464402	G	C	substitution	rs1442532	0.940407	*CNTLN*	NM_017738	INTRON_REGION			-1.18			known common (>5%) SNP, intronic
chr9:20866974-20866974	C	G	substitution	rs7875872	0	*KIAA1797*	NM_017794	EXON_REGION	T718S	2153C>G	2.07	1	58	known SNP, variant found in 100% of reads, change tolerated
chr13:36747798-36747798	C	G	substitution	rs1410633	0.998765	*SOHLH2*	NM_017826	INTRON_REGION			-2.89			known common (>5%) SNP, intronic
chr13:37512624-37512624	G	A	substitution	rs497908	0.91153						1.63			known common (>5%) SNP, non-genic
chr13:37580139-37580139	G	A	substitution	rs1127446	0.340664	*EXOSC8*	NM_181503	EXON_REGION	none	321G>A	2.96			known common (>5%) SNP, nonsynonymous
chr13:37583831-37583831	G	A	substitution	rs9469	0.353545	*FAM48A*	NM_001014286	EXON_REGION	T773M	2318G>A	1.79	0.46	81	known common (>5%) SNP
chr13:37584838-37584838	G	A	substitution	rs7317750	0.83635	*FAM48A*	NM_017569	INTRON_REGION			1.40			known common (>5%) SNP, intronic
chr13:38924083-38924083	A	T	substitution	rs2231330	0.085373	*UFM1*	NM_016617	INTRON_REGION			-1.27			known common (>5%) SNP, intronic
chr13:90644770-90644770	G	A	substitution	rs7982868	0.947218						-2.33			known common (>5%) SNP, non-genic
chr13:90883383-90883383	C	G	substitution	rs12857240	0.538742						-0.51			known common (>5%) SNP, non-genic
chr17:79478019-79478019	G	A	substitution	rs1139405	0.755689	*ACTG1*	NM_001614	EXON_REGION	none	918G>A	1.71			known common (>5%) SNP, nonsynonymous
chr17:80696542-80696542	G	A	substitution	rs2253132	1	*FN3K*	NM_022158	INTRON_REGION			0.26			known common (>5%) SNP, intronic
chr17:80708601-80708601	C	G	substitution	rs1056534	0.372796	*FN3K*	NM_022158	EXON_REGION	none	900C>G	-0.82			known common (>5%) SNP, nonsynonymous
