# Supplementary material for: Exome Sequencing in an Admixed Isolated Population Indicates NFXL1 Variants Confer a Risk for Specific Language Impairment
Source: PLoS Genet. 2015 Mar 17;11(3):e1004925. doi: 10.1371/journal.pgen.1004925 (PMC4363375; doi:10.1371/journal.pgen.1004925)
Supplement: S4 Table — The NFXL1 variant has an expected population frequency of between 0.033 (1000 genomes CLM) and 0.09 (1000 genomes PUR) and is predicted to be present in both founder brothers (frequency in founder brothers of 0.5). Given the population structure, it would therefore be expected to be present in the current population at a frequency of between 0.08 (MAF = 0.03) and 0.14 (MAF = 0.10). Although the frequency of the NFXL1 variant in the founder-related individuals of the Robinson Crusoe validation cohort was at this expected level (0.125), the variant allele was found to cosegregate with language impairment; the frequency of the NFXL1 variant in the founder-related individuals with SLI was above expected (0.194) while that of founder-related individuals with typical language was below expected (0.048), supporting a pathogenic role for this allele. (PDF) [file pgen.1004925.s008.pdf]

Table S4 - Genotype Reconstruction Simulations

Minor allele freq	Frequency of allele A in founder brothers	No. simulated pedigrees	Frequency of allele A in Robinson Crusoe validation cohort
0.01	1	21	0.13
0.01	0.75	88	0.09
0.01	0.5	9862	0.06
0.01	0.25	19492	0.04
0.01	0	970537	0.01
0.02	1	104	0.13
0.02	0.75	414	0.09
0.02	0.5	20298	0.07
0.02	0.25	37999	0.05
0.02	0	941185	0.02
0.03	1	241	0.13
0.03	0.75	893	0.10
0.03	0.5	30547	0.08
0.03	0.25	55364	0.06
0.03	0	912955	0.03
0.04	1	444	0.14
0.04	0.75	1570	0.11
0.04	0.5	40960	0.08
0.04	0.25	72686	0.07
0.04	0	884340	0.04
0.05	1	691	0.14
0.05	0.75	2446	0.12
0.05	0.5	50721	0.09
0.05	0.25	87716	0.07
0.05	0	858426	0.04
0.1	1	2983	0.19
0.1	0.75	9931	0.17
0.1	0.5	102200	0.14
0.1	0.25	153628	0.12
0.1	0	731258	0.09
0.2	1	14410	0.28
0.2	0.75	38020	0.26
0.2	0.5	197983	0.23
0.2	0.25	230404	0.21
0.2	0	519183	0.18

Minor allele freq	Frequency of allele A in founder brothers	No. simulated pedigrees	Frequency of allele A in Robinson Crusoe validation cohort
0.3	1	38362	0.37
0.3	0.75	82096	0.35
0.3	0.5	275992	0.32
0.3	0.25	248655	0.30
0.3	0	354895	0.27
0.4	1	77955	0.46
0.4	0.75	134264	0.44
0.4	0.5	327279	0.41
0.4	0.25	230142	0.39
0.4	0	230360	0.36
0.5	1	140609	0.55
0.5	0.75	187576	0.53
0.5	0.5	344528	0.50
0.5	0.25	187221	0.48
0.5	0	140066	0.45

The *NFXL1* variant has an expected population frequency of between 0.033 (1000 genomes CLM) and 0.09 (1000 genomes PUR) and is predicted to be present in both founder brothers (frequency in founder brothers of 0.5). Given the population structure, it would therefore be expected to be present in the current population at a frequency of between 0.08 (MAF=0.03) and 0.14 (MAF=0.10). Although the frequency of the *NFXL1* variant in the founder-related individuals of the Robinson Crusoe validation cohort was at this expected level (0.125), the variant allele was found to cosegregate with language impairment; the frequency of the *NFXL1* variant in the founder-related individuals with SLI was above expected (0.194) while that of founder-related individuals with typical language was below expected (0.048), supporting a pathogenic role for this allele.
