# Supplementary material for: Exome Sequencing in an Admixed Isolated Population Indicates NFXL1 Variants Confer a Risk for Specific Language Impairment
Source: PLoS Genet. 2015 Mar 17;11(3):e1004925. doi: 10.1371/journal.pgen.1004925 (PMC4363375; doi:10.1371/journal.pgen.1004925)
Supplement: S1 Text — (PDF) [file pgen.1004925.s009.pdf]

Text S1 - Genotype Reconstruction Simulations

Genotype reconstruction simulations allowed us to investigate how the structure of the Robinson Crusoe founder population affects the allele representation in the current Robinson Crusoe validation cohort. One million pedigree files conforming to the Robinson Crusoe pedigree structure (as used for the association analyses of the Robinson Crusoe validation analyses) were generated for ten models including a single SNP of defined minor allele frequency (MAF) (0.01, 0.02, 0.03, 0.04, 0.05, 0.1, 0.2, 0.3, 0.4, 0.5). The resultant pedigrees were classified according to the allelic combinations of the two founder brothers (AA/AA (allele A frequency of 1.0), AA/AB or AB/AA, (allele A frequency of 0.75), AA/BB or BB/AA or AB/AB (allele A frequency of 0.5), AB/BB or BB/AB (allele A frequency of 0.25), BB/BB (allele A frequency of 0.0)).

The resultant frequency of allele A was calculated for the Robinson Crusoe validation cohort (the last two generations of the pedigree). These simulations showed that frequency of the given allele should be consistently and significantly elevated above expected in the Robinson Crusoe validation cohort, even if the Founder brothers only carried 1 copy of allele A. This elevation will be specific to the non-founders in the pedigree. Incoming founder individuals show allele frequencies in line with expected. See Table S4 for details of expected allele frequencies.
